# Molecular Architecture of the Human Mediator–RNA Polymerase II–TFIIF Assembly

**DOI:** 10.1371/journal.pbio.1000603

**Published:** 2011-03-29

**Authors:** Carrie Bernecky, Patricia Grob, Christopher C. Ebmeier, Eva Nogales, Dylan J. Taatjes

**Affiliations:** 1 Department of Chemistry and Biochemistry, University of Colorado, Boulder, Colorado, United States of America; 2 Howard Hughes Medical Institute and the Department of Molecular and Cell Biology, University of California, Berkeley, California, United States of America; Brandeis University, United States of America

## Abstract

The macromolecular assembly required to initiate transcription of protein-coding genes, known as the Pre-Initiation Complex (PIC), consists of multiple protein complexes and is approximately 3.5 MDa in size. At the heart of this assembly is the Mediator complex, which helps regulate PIC activity and interacts with the RNA polymerase II (pol II) enzyme. The structure of the human Mediator–pol II interface is not well-characterized, whereas attempts to structurally define the Mediator–pol II interaction in yeast have relied on incomplete assemblies of Mediator and/or pol II and have yielded inconsistent interpretations. We have assembled the complete, 1.9 MDa human Mediator–pol II–TFIIF complex from purified components and have characterized its structural organization using cryo-electron microscopy and single-particle reconstruction techniques. The orientation of pol II within this assembly was determined by crystal structure docking and further validated with projection matching experiments, allowing the structural organization of the entire human PIC to be envisioned. Significantly, pol II orientation within the Mediator–pol II–TFIIF assembly can be reconciled with past studies that determined the location of other PIC components relative to pol II itself. Pol II surfaces required for interacting with TFIIB, TFIIE, and promoter DNA (i.e., the pol II cleft) are exposed within the Mediator–pol II–TFIIF structure; RNA exit is unhindered along the RPB4/7 subunits; upstream and downstream DNA is accessible for binding additional factors; and no major structural re-organization is necessary to accommodate the large, multi-subunit TFIIH or TFIID complexes. The data also reveal how pol II binding excludes Mediator–CDK8 subcomplex interactions and provide a structural basis for Mediator-dependent control of PIC assembly and function. Finally, parallel structural analysis of Mediator–pol II complexes lacking TFIIF reveal that TFIIF plays a key role in stabilizing pol II orientation within the assembly.

## Abstract 2

The authors perform a cryo-EM study of the 1.9 MDa human Mediator-RNA polymerase II-TFIIF assembly, which reveals the structural organization of the human transcription initiation apparatus.

## Author Summary

Transcription initiation in humans is regulated by a macromolecular complex formed by the RNA polymerase II enzyme (pol II), Mediator, and the general transcription factors TFIIA, TFIIB, TFIID, TFIIE, TFIIF, and TFIIH. Collectively, these factors are known as the Pre-Initiation Complex (PIC). Although the 1.2 MDa Mediator seems to have a major role in regulating assembly and function of the PIC, a structural understanding of the complex has yet to be established. This study outlines a cryo-EM analysis of the Mediator–pol II assembly in the presence or absence of the dimeric TFIIF complex. We observe that TFIIF is required to stably orient the pol II enzyme within the Mediator–pol II assembly, indicating a novel structural role for TFIIF in transcription initiation. Additionally, we accurately dock the pol II crystal structure within the human Mediator–pol II–TFIIF cryo-EM map. The locations of TFIIH, TBP (a subunit within TFIID), TFIIA, TFIIB, TFIIE, and TFIIF relative to the pol II enzyme itself have been determined by previous studies. These data in combination with the Mediator–pol II–TFIIF structure described here allow us to propose the structural organization of the entire 3.5 MDa human PIC.

## Introduction

In humans, the transcription initiation machinery consists of Mediator, pol II, TFIIA, TFIIB, TFIID, TFIIE, TFIIF, and TFIIH and approximates 3.5 MDa in size. This large assembly can exist in various structural and functional states [1]. When not in an activated state that supports transcription initiation, this assembly is best described as a Pre-Initiation Complex (PIC) [2]. At 1.2 MDa, Mediator represents a major component within the human PIC and based upon biochemical assays, Mediator helps assemble and stabilize the PIC [3],[4]. Human Mediator is known to functionally interact with most PIC components, including TFIIB, TFIID, TFIIE, TFIIH, and pol II itself [3],[5]–[9]. Although Mediator appears critical for controlling the assembly and activity of the PIC, a structural basis for these observations has not been established.

Structural analysis of the human transcription initiation machinery has been hindered by several factors, including the large size and complexity of the machinery itself. Although it is well-established from biochemical assays that pol II physically interacts with human Mediator [5],[7], little is known about the pol II–Mediator interface. Attempts to structurally define the pol II–Mediator interface have been made in yeast, but these have been limited to incomplete assemblies of Mediator and/or pol II [10],[11]. Perhaps as a consequence, these studies have provided inconsistent predictions of pol II orientation relative to Mediator itself [12],[13]. Because structural data are not available for the complete Mediator–pol II assembly, even the most basic information about human PIC structure remains unknown, such as how PIC components might assemble together with Mediator at a promoter. For instance, it is not known how pol II orients upon interaction with Mediator. Because the location of other PIC factors (TFIIA, TFIIB, TBP, TFIIE, TFIIF, TFIIH) has been determined relative to pol II itself [14]–[18], identifying the pol II orientation when bound to Mediator would help define the structural organization of the entire 3.5 MDa human PIC. Thus, structural analysis of the Mediator–pol II assembly represents an essential, yet missing, link to defining the molecular architecture of the human PIC. It is also unclear how the interaction of Mediator and pol II permits simultaneous assembly of the large, 1.1 MDa TFIID complex as well as other PIC components—such as TFIIB, TFIIE, and TFIIF—that interact directly with pol II during transcription initiation. Finally, Mediator is required for TFIIH-dependent pol II CTD phosphorylation within the human PIC [9], yet it is not established how the pol II CTD might track within the PIC, nor is it known what structural features within the Mediator–pol II assembly could allow for regulation of TFIIH-dependent pol II CTD phosphorylation.

The large size, low-abundance, and dynamic features of the human Mediator complex prevent an analysis using high-resolution techniques such as X-ray crystallography or NMR spectroscopy. However, structural analysis of Mediator is well-suited for cryo-EM studies, which require sub-microgram quantities of purified protein and can potentially resolve alternate conformational states of macromolecular complexes. We purified two different sub-assemblies within the 3.5 MDa PIC: the 1.8 MDa Mediator–pol II binary complex and the 1.9 MDa Mediator–pol II–TFIIF assembly. In each case, Mediator was bound to the activation domain of VP16. Cryo-EM analysis of each assembly revealed the overall structural organization of the entire human PIC and identified a role for TFIIF in stabilizing Mediator–pol II interactions. Our results establish Mediator as the scaffold around which the entire human PIC assembles and reveal a pol II-induced structural shift within Mediator that likely precludes Mediator binding to the CDK8 submodule. Collectively, these observations provide a structural basis for initiation and post-initiation regulatory events and further define how Mediator coordinates PIC assembly and function.

## Results

### Isolation of Human Mediator–pol II–TFIIF or Mediator–pol II Complexes

**Figure 1:**
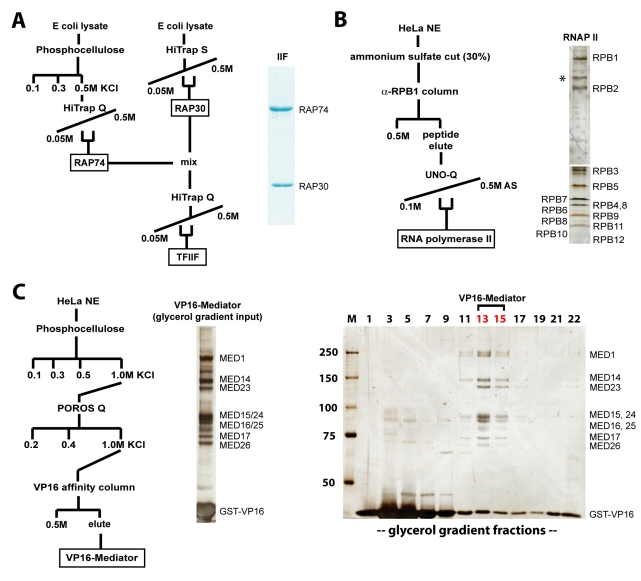
Purification of factors used in this study. (A) Schematic outlining the purification of human TFIIF and a Coomassie stained gel of the resulting purified material. (B) Schematic outlining the purification of endogenous human pol II and a silver stained gel of the purified complex. * Probable breakdown product of RPB1. This band is recognized by RPB1 antibodies in immunoblot experiments and its presence increases in proportion to the number of times the purified pol II sample is freeze-thawed. Pol II utilized for EM analysis was not freeze-thawed, whereas the silver-stained pol II sample shown here was freeze-thawed once. (C) Purification overview for human VP16-Mediator and a silver stained gel of the purified material. Also shown is a glycerol gradient purification of this sample (right). Mediator-containing fractions (13–15) that were subjected to MS analysis (Table 1) are highlighted.

In order to assemble the Mediator–pol II–TFIIF complex or the Mediator–pol II binary complex, we first purified Mediator, pol II, and TFIIF independently. Human TFIIF was purified following recombinant expression in *E. coli*, whereas pol II and Mediator were each isolated as endogenous complexes from HeLa cells. Mediator purification involved an affinity resin using the activation domain of VP16 (residues 411–490), yielding VP16-bound Mediator complexes [19]. Each complex (TFIIF, VP16-Mediator, and pol II) was purified to near-homogeneity, as shown in Figure 1A–C. We completed mass spectrometry analysis of Mediator and pol II, primarily to confirm that these purified complexes contained each of their consensus subunits: 26 subunits within Mediator and 12 subunits for pol II (Table 1). With the purified, 26-subunit Mediator complex and the 12-subunit pol II complex in hand, we next tested whether Mediator and pol II and/or Mediator, pol II, and TFIIF would associate to form a stable assembly that could be isolated and imaged using electron microscopy.

**Table 1:** Mass spectrometry data for human pol II (A) and human Mediator (B) samples.

	A	
Approx. MW (KDa)	Pol II Subunit	Percent Coverage
220	RPB1	29.2
130	RPB2	32.7
35	RPB3	25.5
17	RPB4	40.8
26	RPB5	29.5
18	RPB6	8.7
22	RPB7	35.5
17	RPB8	46.7
15	RPB9	67.2
12	RPB10	14.2
10	RPB11	14.2
8	RPB12	16.4

	B	
Approx. MW (KDa)	Mediator Subunit	Percent Coverage
220	MED1	14.4
36	MED4	17.0
33	MED6	24.8
34	MED7	15.0
32	MED8	19.9
16	MED9	11.6
16	MED10	9.6
13	MED11	23.9
150	MED14	11.3
105	MED15	13.6
95	MED16	8.3
78	MED17	10.3
28	MED18	11.1
26	MED19	4.5
23	MED20	16.0
19	MED21	12.5
16	MED22	21.4
130	MED23	4.3
100	MED24	7.9
92	MED25	5.8
70	MED26	18.3
37	MED27	3.5
20	MED28	11.8*
24	MED29	26.7
25	MED30	14.0
18	MED31	26.7

A mass spectrometry (MS) protocol was used to define the composition of Mediator and pol II that was used for EM analysis. These data confirm the purification protocols outlined in Figure 1B and Figure 1C isolated the complete, 26-subunit Mediator complex and the entire, 12-subunit pol II enzyme. Peptide identification and percent coverage was calculated following a 1% false-discovery rate (FDR) analysis.

*No unique peptides corresponding to MED28 remained following the 1% FDR screen; however, two unique MED28 peptides were present in the MS data and these were manually validated and are shown in Figure S11. The 11.8% value listed for MED28 reflects the inclusion of these two peptides. Thus, it does not appear that MED28 dissociates from Mediator during the purification protocol outlined in Figure 1C. Note also that whereas CDK8 and Cyclin C were not detected in this sample, MED12 (2.6% coverage) and MED13 (0.8% coverage) were detectable; based upon the silver stained gel of VP16-Mediator (Figure 1C) as well as comparison of spectral counts and percent coverage, it is evident these subunits are substantially sub-stoichiometric relative to core Mediator subunits in this VP16-Mediator sample.

**Figure 2:**
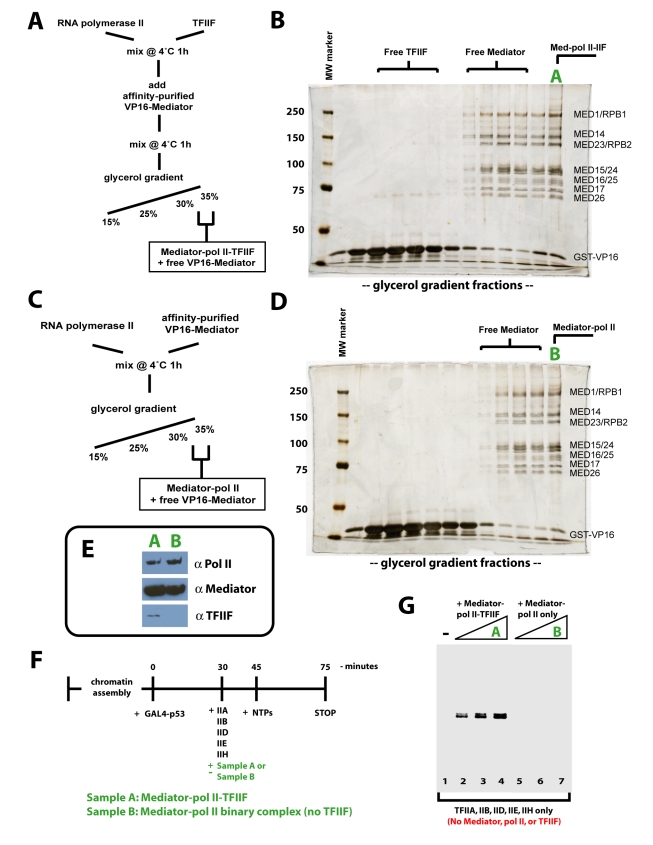
Purification and activity of Mediator–pol II–TFIIF or the Mediator–pol II binary complex. (A) Schematic outlining the isolation of the Mediator–pol II–TFIIF assembly from individually purified components. Note Mediator was purified bound to the VP16 activation domain (residues 411–490). (B) Silver stained polyacrylamide gel of glycerol gradient fractions from the purification outlined in (A). Subunits are labeled at right. Fraction A denotes the fraction containing the 1.9 MDa Mediator–pol II–TFIIF assembly. (C) Purification scheme used for isolation of the Mediator–pol II binary complex. (D) Silver stained gel of the odd fractions of the glycerol gradient resulting from the purification outlined in (C). Mediator and pol II subunits are listed at the right. Fraction B denotes the sample containing Mediator–pol II. (E) Western blot analysis of pol II (anti-RPB1), Mediator (anti-MED23), and TFIIF (anti-Rap74), which confirms the presence of TFIIF in fraction A and the absence of TFIIF in fraction B. (F) Schematic of the transcription assay used to investigate the activity of the isolated Mediator–pol II assemblies. Note that Mediator, pol II, and TFIIF are excluded from these assays. (G) The isolated Mediator–pol II–TFIIF assembly is transcriptionally active. In vitro transcription indicates the isolated Mediator–pol II–TFIIF assembly (fraction A) can reconstitute activated transcription from reactions lacking these factors, whereas a Mediator–pol II assembly lacking TFIIF cannot (fraction B).

To isolate the Mediator–pol II–TFIIF assembly, pol II and TFIIF (added in excess to pol II) were incubated together for 1 h at 4°C. Mediator was then added and all three factors were incubated together for an additional hour at 4°C. After incubation, the sample containing Mediator, pol II, and TFIIF was loaded onto a glycerol gradient (Figure 2A). The gradient was designed such that the complete, 1.9 MDa Mediator–pol II–TFIIF assembly would migrate and concentrate within the final 1–2 fractions, whereas free Mediator or pol II would mostly sediment within earlier gradient fractions (Figure 2B and unpublished data). As a 100 kDa dimer, free TFIIF sedimented much earlier in the gradient (Figure 2B). The presence of Mediator, pol II, and TFIIF within the final gradient fraction—denoted fraction A—was confirmed by immunoblotting experiments (Figure 2E). Because TFIIF alone sediments much earlier than fraction A, this immunoblotting result provided biochemical evidence that Mediator, pol II, and TFIIF were forming a stable, ternary complex.

To investigate the potential impact of TFIIF on the Mediator–pol II structure, we also isolated a Mediator–pol II binary complex using a method similar to that described for isolation of Mediator–pol II–TFIIF (Figure 2C). The glycerol gradient corresponding to the Mediator–pol II experiment showed a silver-stain pattern consistent with the presence of both Mediator and pol II in the last fraction (fraction B, Figure 2D), as expected. Western blot experiments confirmed the presence of Mediator and pol II in this fraction, whereas TFIIF—which was not added in this protocol—was not detected (Figure 2E).

To further confirm that TFIIF was present together with Mediator and pol II in gradient fraction A (Figure 2B), but absent in gradient fraction B (Figure 2D), we used an in vitro transcription assay consisting of purified and recombinant human factors [20]. Because this assay requires reconstitution of the transcription machinery from purified components, transcription initiation will not occur if a PIC component (e.g. TFIIF) is not added to the reaction. An outline of the transcription assay is shown in Figure 2F. Following addition of activator (GAL4-p53), the general transcription factors TFIIA, IIB, IID, IIE, and IIH were added to chromatin templates, together with fraction A or fraction B. As shown in Figure 2G, transcription reactions that were not supplemented with a glycerol gradient fraction (and therefore lacked Mediator, pol II, and TFIIF) were inactive, as expected (lane 1). Upon supplementation with gradient fraction A, transcription was activated in a dose-dependent manner (lanes 2–4). By contrast, supplementation with gradient fraction B, which contains Mediator and pol II but lacks TFIIF, was unable to support transcription (lanes 5–7). Taken together, the data in Figure 2G further demonstrated TFIIF was present with Mediator and pol II within glycerol gradient fraction A, whereas TFIIF was absent from glycerol gradient fraction B.

### Initial EM Analysis of the Mediator–pol II–TFIIF Assembly

Given the functional (Figure 2G) and biochemical (Figure 2E) evidence that Mediator, pol II, and TFIIF formed a stable assembly, we next examined this sample (fraction A) using EM. We first imaged negatively stained samples and collected both untilted (0°) and tilted (25°–45°) images to produce an *ab initio* random conical tilt reconstruction [21]. Examination of untilted micrographs revealed a relatively homogenous field of particles with a size and shape consistent with an intact, 1.9 MDa Mediator–pol II–TFIIF assembly (Figure S1A). Reference-free 2D classification followed by back-projection and cross-correlation of the corresponding 3D model structures established a homogenous data set that represented 36% of all single-particle images. This data set was then used to generate an initial reference volume, which was subjected to iterative projection matching [22] to produce a final, refined structure at 42 Å resolution (Figure S1B–D). A number of reprojections of the refined, Mediator–pol II–TFIIF structure are compared with those of Mediator alone in Figure S1E to highlight the significant change in protein density within the Mediator head region. Based upon this comparison, it was evident that pol II interacts with the Mediator head domain, as observed previously with yeast Mediator–pol II complexes [10],[23]. This Mediator–pol II–TFIIF structure, obtained from negatively stained samples using the random conical tilt methodology, served as a starting point for cryo-EM refinement of Mediator–pol II–TFIIF assemblies.

### Cryo-EM Reconstruction of Mediator–pol II–TFIIF

Our studies using negatively stained samples indicated that VP16-Mediator, pol II, and TFIIF could form a stable assembly that was amenable to 3D reconstruction using single-particle methods. We next obtained EM data from the same sample (i.e. fraction A, Figure 2B) using cryo-EM techniques, which allows for samples to be imaged in a fully hydrated state, offering the potential for higher resolution structural information and implementation of a powerful 3D variance technique [24] to assess potential structural variability within the sample.

Cryo-EM micrographs of Mediator–pol II–TFIIF were collected and screened for astigmatism and sample stage drift (see Materials and Methods). The best 106 micrographs were selected, from which 10,856 single-particle images were obtained for image processing. A representative micrograph and its corresponding power spectrum is shown in Figure S2A and S2B. The initial Mediator–pol II–TFIIF structure, generated from negatively stained samples, was low-pass filtered to 57 Å resolution and used as an initial reference volume for iterative projection matching refinement. Initial refinement with the cryo-EM data improved the structure, but the resolution leveled off at around 48 Å. This suggested some conformational or compositional heterogeneity within the cryo-EM data set. Such heterogeneity was in fact expected, as VP16-Mediator was added in excess of pol II during the isolation of the Mediator–pol II–TFIIF assembly and a fraction of complexes within the cryo-EM data set should correspond to free VP16-Mediator.

**Figure 3:**
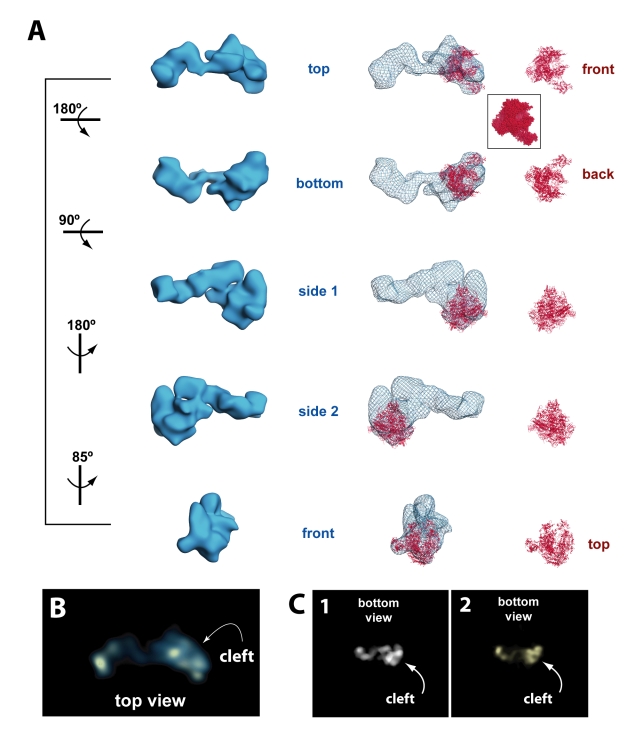
RNA polymerase II adopts a stable orientation within the Mediator–pol II–TFIIF assembly. (A) Left: different views (rotation shown at left) of the cryo-EM 3D reconstruction of the Mediator–pol II–TFIIF assembly, rendered to 1.8 MDa; center: the pol II crystal structure (red; PDB 1Y1V) is shown docked within the Mediator–pol II–TFIIF cryo-EM map (blue mesh); right: the pol II crystal structure displayed on its own, with characteristic pol II orientations denoted in red font. Note the docked pol II crystal structure shows only the polypeptide backbone and does not correspond to the overall electron density map, whereas the cryo-EM map represents electron density (i.e. a space-filling rendering). A space-filling model of the pol II “front” view is shown (inset) for reference. (B) The top view of the Mediator–pol II–TFIIF assembly cryo-EM structure rendered in the “solid” view using Chimera [59]. Bright areas indicate higher protein density. This rendering allows clear visualization of the pol II cleft. (C) A reprojection view (panel 1) of the Mediator–pol II–TFIIF 3D reconstruction that further highlights the location of the pol II cleft in the structure; panel 2 shows Mediator–pol II–TFIIF in the same orientation, rendered in the “solid” view using Chimera.

To partition the cryo-EM data into distinct complexes we carried out multi-reference refinement using as references the negative stain reconstructions of the free VP16-Mediator structure [19] and the Mediator–pol II–TFIIF assembly structure, each filtered to 57 Å (see Materials and Methods). Using this protocol, individual cryo-EM images were aligned to re-projections of each distinct reference (VP16-Mediator or Mediator–pol II–TFIIF) and partitioned to the structure that yielded the highest cross-correlation. In this way, the cryo-EM data set was separated into two, more homogenous groups. The free VP16-Mediator cryo-EM structure that resulted from this refinement is shown in Figure S3, whereas different views of the Mediator–pol II–TFIIF assembly are shown along the left panel of Figure 3A (see also Movie S1). Importantly, separation of free VP16-Mediator images from Mediator–pol II–TFIIF significantly improved the resolution of the Mediator–pol II–TFIIF reconstruction from 48 Å to 36 Å (Figure S2C), based upon the FSC criterion (or 26 Å using the 3σ criterion). The distribution of particle orientations within the Mediator–pol II–TFIIF data set was fairly isotropic, although proportionally fewer end-on views were observed (Figure S2D).

To further assess the quality of the Mediator–pol II–TFIIF cryo-EM 3D reconstruction, we generated reference-free 2D class averages from the cryo-EM data set, using a k-means clustering algorithm [25]. As expected, 2D classes resembling Mediator–pol II–TFIIF or the free VP16-Mediator structure were observed. These 2D class averages—generated without any reference bias—were then compared with 2D class averages derived from re-projections of the Mediator–pol II–TFIIF assembly shown in Figure 3A. As shown in Figure S4A, the reference-free 2D class averages closely matched reference-based 2D class averages derived from re-projections of the refined Mediator–pol II–TFIIF structure, supporting the validity of the cryo-EM reconstruction. Similarly, the reference-free 2D class averages closely matched 2D class averages derived from re-projections of the free VP16-Mediator structure (Figure S4B). We also completed protocols to ensure that refinement of Mediator–pol II–TFIIF was not negatively impacted by model bias [26]; these experiments are described in Materials and Methods. Additional strategies were implemented to further refine the Mediator–pol II–TFIIF cryo-EM structure; however, improvement of the resolution beyond 36 Å was not achieved, likely because of flexibility inherent within the 1.9 MDa assembly (see below). Importantly, the 36 Å resolution assembly structure is sufficient for accurate pol II docking studies (Figure S5).

### RNA Polymerase II Orientation Within the Mediator–pol II–TFIIF Assembly

We utilized two complementary approaches to determine the orientation of pol II within the Mediator–pol II–TFIIF assembly. We began by docking a crystal structure of yeast pol II into the cryo-EM density map using the program Situs [27]. Docking pol II with Situs represents a rigorous, unbiased way in which to probe pol II orientation, and regardless of where the pol II crystal structure was initially positioned (e.g. outside the cryo-EM map or within the leg domain or within the head domain), Situs calculated the same docking result. Rigid body docking of the pol II atomic model indicated a best fit within the Mediator head region (Figure 3A, center panels; see also Movie S1), as expected based on comparison of the structure with that of Mediator alone. Note the pol II atomic model shown throughout this article is PDB 1Y1V [28]. This model was chosen because it most closely matches the human pol II structure determined by cryo-EM [29]. However, docking calculations completed with over a dozen distinct pol II crystal structures yielded the same results (Table S1). The orientation of the pol II cleft in this docking model is perpendicular to the long axis of Mediator such that downstream DNA would extend from the “top” of the assembly. This pol II orientation can readily accommodate binding of other PIC factors (see Discussion). As an alternate means of determining the orientation of pol II within the Mediator–pol II–TFIIF assembly, we used a projection matching strategy (see Materials and Methods) in which 2D projections of the human pol II cryo-EM structure [29] were aligned and cross-correlated with 2D projections of the human Mediator-pol II-TFIIF assembly. This independent analysis resulted in the same pol II orientation within the Mediator–pol II–TFIIF assembly as that calculated by docking of the yeast pol II crystal structure (Figure S6).

Given the consistent pol II docking calculations, corroborated by the projection matching data, it was evident that pol II adopted a stable orientation within the Mediator–pol II–TFIIF assembly. The pol II orientation calculated from these alternate methods indicated the pol II cleft was exposed at one end of the assembly (Figure 3A). Because 2D projections of a 3D volume allow an assessment of protein density throughout the volume, 2D projection views from the “top” and “bottom” of the Mediator–pol II–TFIIF structure should provide an additional means to probe the location and orientation of the pol II cleft. As shown in Figure 3B and Figure 3C, the 2D projection views reveal an area deficient in protein density that overlaps precisely with the pol II cleft, offering an additional verification of the pol II docking and projection matching results.

The cryo-EM structure of the entire Mediator–pol II–TFIIF assembly shown in Figure 3A was reconstructed using 46% of the data. Single-particle images were included in the reconstruction based upon a cross-correlation threshold. Two additional 3D reconstructions were completed *de novo* in which a greater percentage of the cryo-EM data was included, based upon adjusting the cross-correlation threshold (see Materials and Methods). As before, the multi-reference refinement protocol was implemented. Single-particle images were free to align to reference projections derived from either of the major entities present in the sample: the free VP16-Mediator structure or the Mediator–pol II–TFIIF complex. In each case (62% or 55% of the data was included in the analysis, instead of 46%), the Mediator–pol II–TFIIF assembly refined to essentially the same structure as shown in Figure 3A, including an identical docking solution for pol II. However, the resolution did not improve, and small areas of structural discontinuity became evident with the larger data sets, likely due to inclusion of alternate conformational states. These results suggested that inherent flexibility within the Mediator–pol II–TFIIF assembly was limiting the ultimate resolution of the reconstruction. In support of this, we further probed for alternate Mediator–pol II–TFIIF structural states using 3D variance analysis [24], which yielded no structure distinct from that shown in Figure 3A (see Materials and Methods).

A comparison of the free VP16-Mediator structure (Figure S3 and [19]) with that of the Mediator–pol II–TFIIF assembly indicates that Mediator itself undergoes significant structural shifts upon binding pol II–TFIIF. Structural shifts occur not only at the Mediator–pol II interface, but also throughout the complex, including the Mediator leg domain (Figure S7). As a consequence, difference map calculations (e.g. VP16-Mediator with or without bound pol II–TFIIF) are not informative. Despite the limited sequence conservation between yeast and human Mediator (Table S2), it is notable that global structural shifts are also observed upon pol II binding to yeast Mediator [10]. A scheme outlining human VP16-Mediator structural shifts that occur upon pol II–TFIIF binding is shown in Figure S7.

### Initial EM Analysis of the Mediator–pol II Binary Complex

Upon completion of the cryo-EM reconstruction of Mediator–pol II–TFIIF, we next analyzed the Mediator–pol II sample (Figure 2D, fraction B) using EM. As with the Mediator–pol II–TFIIF assembly, EM analysis of fraction B began with analysis of negatively stained samples to generate an initial Mediator–pol II structure. As expected, extra density in this structure was apparent within the head domain of Mediator, indicating that pol II associates with the Mediator head domain even in the absence of TFIIF. A description of the EM image processing of the Mediator–pol II data with random conical tilt, negatively stained samples is provided in Materials and Methods.

### Cryo-EM Reconstruction of Mediator–pol II Reveals Key Structural Role for TFIIF

The 3D reconstruction of the Mediator–pol II binary complex obtained from negatively stained samples was used as a starting model for cryo-EM analysis of the complex 141 cryo-micrographs, screened for astigmatism and sample drift, were used (Figure S8A). As with the Mediator–pol II–TFIIF cryo-EM reconstruction, image processing was initiated using a multi-reference approach. The low-pass filtered (to 57 Å) free VP16-Mediator structure [19] and the Mediator–pol II structure (generated from negatively stained samples) were used as initial references. Throughout the refinement it was evident the Mediator–pol II data set was more structurally heterogeneous than the Mediator–pol II–TFIIF sample (see Materials and Methods). As a result, initial refinements of Mediator–pol II contained regions of discontinuity, suggesting the presence of multiple conformational states within the Mediator–pol II data set. By contrast, data that partitioned to the free VP16-Mediator structure—the second volume included in this initial multi-reference protocol—refined normally: its resolution improved throughout the refinement and its structure matched the previously published structure of VP16-Mediator [19].

**Figure 4:**
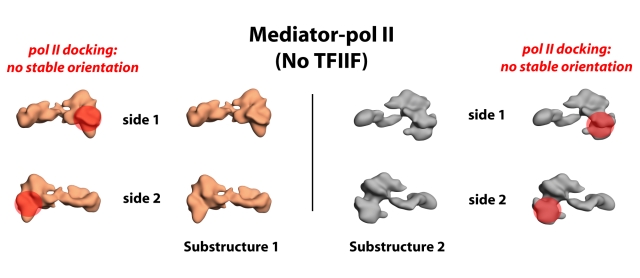
Pol II does not stably orient within Mediator in the absence of TFIIF. The two distinct Mediator–pol II substructures are shown. The location of pol II within each structure—based upon docking the pol II crystal structure (PDB 1Y1V) in Situs [27]—is denoted by the orange sphere. The orientation of pol II, however, could not be reliably determined from docking or projection matching calculations; it appears that multiple pol II orientations exist in the absence of TFIIF.

To probe for additional conformational states within the Mediator–pol II cryo-EM data, we implemented a 3D variance and focused classification procedure [24]. This identified a region of peak structural variance near the pol II binding site (region 1, Figure S9). Cryo-EM images within the Mediator–pol II data set were then sorted into two groups based on focused classification within this region (see Materials and Methods). Two new Mediator–pol II reference structures that resulted from this classification were then used for angular refinement against the cryo-EM data set. Thus, a new multi-reference angular refinement was completed that partitioned the data into one of three reference volumes: free VP16-Mediator, or two distinct Mediator–pol II substructures. Structure refinement improved substantially with this revised multi-reference procedure. In particular, each of the two Mediator–pol II substructures refined to an improved resolution (34 Å for substructure 1; 36 Å for substructure 2), and structure discontinuity was eliminated. The 3D reconstruction of each Mediator–pol II structure is shown in Figure 4; see also Figure S8B–E.

To define the orientation of pol II within each Mediator–pol II structural state, docking experiments were completed using Situs [27]. In contrast to the Mediator–pol II–TFIIF structure, however, a high-confidence docking result could not be attained for either Mediator–pol II complex (substructure 1 or substructure 2). Although pol II localized consistently to the Mediator head domain region in each substructure, its orientation was variable and undefined (Figure 4). Projection matching experiments provided similar results in that a defined, stable pol II orientation was not evident for Mediator–pol II substructure 1 or substructure 2 (unpublished data). Because the same Mediator sample and the same pol II sample were used to assemble both Mediator–pol II–TFIIF and Mediator–pol II (see Materials and Methods), it appears the absence of TFIIF is solely responsible for the dramatically different structure of the Mediator–pol II complex relative to Mediator–pol II–TFIIF. From these data, we conclude that pol II can associate with Mediator in the absence of TFIIF, but pol II does not adopt a stable orientation. By contrast, pol II does adopt a stable orientation within the Mediator–pol II–TFIIF assembly, suggesting TFIIF helps orient and stabilize pol II when bound to Mediator.

## Discussion

Although the proteins and protein complexes required to regulate transcription initiation have been known for some time, an understanding of how the initiation machinery assembles and functions as a unit has remained elusive. One reason for this is that PIC structure has not been examined in the context of the major architectural factor within the PIC—the human Mediator complex. The cryo-EM study outlined here involved a stable assembly of three different multi-subunit complexes: the 26-subunit Mediator complex, the 12-subunit pol II complex, and the dimeric TFIIF complex. We observed that the Mediator–pol II–TFIIF ternary complex was more stable than the Mediator–pol II binary complex. Whereas cross-linking was not required to assemble the ternary complex, TFIIF was clearly important for stabilizing the orientation of pol II within the assembly. This observation defines a structural role for TFIIF within the PIC that likely contributes to its requirement for transcription initiation. We propose that TFIIF makes simultaneous contacts with both Mediator and pol II to stabilize the assembly; however, further experiments will be required to confirm this.

The human Mediator complex is large and structurally dynamic. Not only does pol II binding induce structural shifts in Mediator, but activator binding or CDK8 submodule binding also triggers substantial structural shifts throughout the complex [30]. Structural plasticity is also inferred from bioinformatics studies that predict an unusually high percentage of intrinsically disordered regions within Mediator subunits [31]. This structural flexibility appears to be essential for the biological activity of the human Mediator complex [9],[32]. We employed numerous image processing strategies that partitioned the Mediator–pol II–TFIIF cryo-EM data into groups with improved structural homogeneity. Although successful, the ultimate resolution of the assembly did not exceed 36 Å (0.5 FSC criterion, or 26 Å using the 3σ resolution assessment). A cryo-EM study of the 21-subunit yeast Mediator complex resulted in a 28 Å structure (0.5 FSC criterion), and a cryo-EM study of the 12-subunit human pol II enzyme yielded a 22 Å resolution structure [12],[29]. As with the yeast Mediator cryo-EM study, structural flexibility within the pol II enzyme was cited as a limiting factor to obtaining higher resolution. As a 1.9 MDa assembly of three distinct protein complexes containing 40 subunits, human Mediator–pol II–TFIIF represents the largest transcription assembly ever examined using cryo-EM. Because of the dynamic nature of the complexes within this assembly, we anticipate that chemical fixation or crystallization will be required to obtain structural information that is substantially higher resolution.

Given the importance of defining the correct orientation of pol II within the Mediator–pol II–TFIIF assembly, we implemented a series of different techniques to confirm the docking result. First, identical docking results were obtained from independent EM data sets, including the random conical tilt negative stain data and the cryo-EM data. Second, three different, *de novo* cryo-EM reconstructions yielded essentially identical structures with the same pol II docking result. Third, identical pol II docking results were calculated from over 20 different pol II crystal structures (e.g. PDB 1Y1V, 1NT9, etc.). Fourth, pol II projection matching experiments, which represent a completely distinct means of evaluating pol II orientation, yielded a result consistent with the pol II docking experiments. Fifth, features resembling the pol II stalk and cleft are clearly visualized within the refined cryo-EM map and the locations of these features coincide precisely with the pol II crystal structure docking results. Finally, and perhaps most compelling, the calculated orientation of pol II within the Mediator–pol II–TFIIF assembly can be reconciled with existing biophysical studies that localized other GTFs within the PIC relative to pol II itself (see below).

### Structural Model of the Complete, 3.5 MDa Human PIC

**Figure 5:**
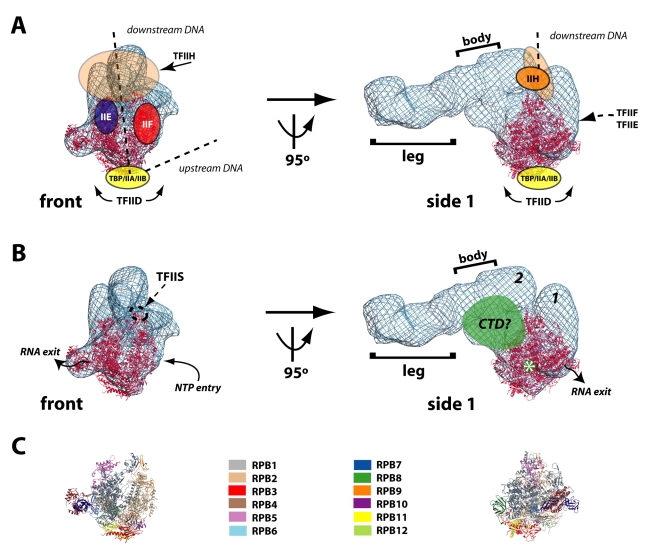
Structural model of the entire, 3.5 MDa human PIC. (A) The “front” and “side 1” views of the Mediator–pol II–TFIIF cryo-EM map (blue mesh) are shown. The docked pol II enzyme is shown in red. The locations of TBP, TFIID, TFIIA, TFIIB, TFIIF, TFIIE, and TFIIH are superimposed upon the cryo-EM map and are based upon existing crystallography, EM, and cross-linking studies [14]–[18],[36]. The likely path of upstream and downstream promoter DNA is also shown (dashed line). (B) The binding site for TFIIS is shown, along with the NTP entry and RNA exit sites within the assembly [28],[37]. The putative location of the pol II CTD is shown in green (see text). The Mediator densities labeled “1” and “2” correspond to the head domain. The asterisk denotes the site from which the pol II CTD extends from the enzyme. (C) Ribbon diagram of pol II alone, shown in the same orientation as in (B). Individual pol II subunits colored as shown.

Several labs have used X-ray crystallography or crosslinking experiments to determine where the general transcription factors TFIIA, TFIIB, TBP, TFIIE, TFIIF, and TFIIH reside in the PIC relative to pol II itself [14]–[18],[33],[34]. Because pol II adopts a defined orientation within the Mediator–pol II–TFIIF assembly, the structural data from previous studies can now augment the Mediator–pol II–TFIIF cryo-EM map. By merging these data, the structural organization of the entire human PIC can be proposed (Figure 5A). Importantly, the location and orientation of pol II within the cryo-EM assembly is completely consistent with previous structural studies that examined pol II and other general transcription factors. For example, surfaces along pol II shown to be required for interaction with TFIIB and TFIIE are exposed and accessible in the Mediator–pol II–TFIIF assembly [15]–[17]. Furthermore, no significant structural rearrangements appear necessary to accommodate the large, multi-subunit TFIID and TFIIH complexes (Figure S10) [35],[36]. The pol II cleft, which interacts with promoter DNA, is also accessible in the Mediator–pol II–TFIIF assembly and an unobstructed path for the upstream and downstream DNA can be envisioned (Figure 5A). Along the surface of the pol II subunit RPB2, the entry site for NTPs (the pore and funnel) is accessible as is the docking site for potential interactions with TFIIS (Figure 5B) [28],[37].

The fact that previous structural models of pol II together with TFIIB, TFIID, TFIIE, or TFIIH can be incorporated within the human Mediator–pol II–TFIIF cryo-EM map offers further validation of the cryo-EM structure and the pol II docking results. By contrast, these same structural models are not compatible with the various structures proposed for yeast Mediator-yeast pol II (Figure S10) [10],[11],[13]. At least four factors might contribute to the differences observed between the human and yeast PIC models. First and foremost, the cryo-EM study outlined here involves the entire, 26-subunit human Mediator complex and the complete, 12-subunit human pol II enzyme. No structural study in yeast has examined the entire 12-subunit pol II enzyme together with Mediator; moreover, pol II docking calculations—which represent the most rigorous, unbiased means to determine pol II orientation within an EM map—have not been completed with yeast factors. One yeast PIC model is based upon a recent EM study that examined a 7-protein yeast Mediator head module and two subunits of yeast pol II, RPB4 and RPB7 [11]. Yet biochemical experiments indicate the 7-subunit yeast head module does not interact with the pol II CTD, nor can it stably interact with the entire pol II enzyme [38]; additional Mediator subunits are required for pol II binding. The pol II–Mediator interface is extensive and clearly involves more than the 7-subunit Mediator head module and does not require the RPB4/7 subunits [10],[23]. Consequently, an alternate pol II orientation proposed from EM analysis of the 7-subunit yeast Mediator and RPB4/7 dimer likely derives from the fact that interactions required for stable pol II–Mediator binding could not occur [11]. Further supporting this idea, pol II orientation within the yeast pol II-yeast Mediator assembly appears to shift substantially when the entire yeast Mediator complex is examined together with a 10-subunit pol II enzyme that lacks RPB4/7 [10],[23]. Modeling the RPB4/7 stalk into the EM structure of yeast Mediator bound to the 10-subunit pol II enzyme orients RPB4/7 toward the middle/tail domain of yeast Mediator [13], whereas the study with the 7-subunit yeast head module proposes that RPB4/7 physically interacts with the Mediator head domain [11]. A second potential reason for the structural differences proposed for yeast Mediator-pol II complexes is that structural studies in yeast have not examined Mediator and pol II together with TFIIF; the structural data outlined here indicate TFIIF plays a major role in orienting human pol II relative to Mediator itself. Third, our structural studies examined human Mediator bound to the activation domain of VP16, whereas structural studies with yeast Mediator and yeast pol II did not examine Mediator bound to any activation domain. Lastly, whereas pol II and several other general transcription factors (e.g. TFIIB, TFIIE, TFIIF) are relatively well-conserved, Mediator is poorly conserved between yeast and humans (Table S2). Thus, it is plausible that the human transcription initiation machinery may adopt a distinct architecture relative to that coordinated by yeast Mediator. A summary comparing structural studies with yeast and human Mediator–pol II complexes is shown in Table 2.

**Table 2:** Comparison of EM studies with Mediator and RNA polymerase II.

Organism	Yeast	Yeast	Human	Human
**Composition**	Mediator (21 subunits)	Mediator (7 subunits)^c^	Mediator (26 subunits)	Mediator (26 subunits)
	Pol II (10 subunits)^b^	RPB4 and RPB7	Pol II (12 subunits)	Pol II (12 subunits)
			TFIIF (2 subunits)	
**EM method**	negative stain	negative stain	cryo-negative stain	cryo-negative stain
**Initial data set**	∼3,000 particles	∼8,000 particles	10,856 particles^e^	7,962 particles^e^
**Final data set** a	393 particles	1,332 particles	4,994–6,731 particles^d^	1,687 particles and 1,990 particles^f^
**Resolution**	∼35 Å	30–35 Å	36 Å	34 Å and 36 Å
**Reference**	10	11	This study	This study

a The number of single-particle images included in the final angular refinement: the structurally homogenous data set.

b Pol II in this sample was missing RPB4 and RPB7.

c Yeast Mediator head module.

d Different subsets of the data could be used that yielded similar structures with identical pol II docking results.

e Particle number includes free VP16-Mediator, which was also present in these samples and was refined independently (e.g. see Figure S3).

f Two distinct Mediator–pol II substructures were observed in the absence of TFIIF.

### Transcription Initiation and Elongation

The human pol II enzyme contains a C-terminal domain (CTD) within its RPB1 subunit that is approximately 500 residues in length and is largely unstructured. The pol II CTD interacts with the human Mediator complex; in fact, the pol II CTD can be used to affinity purify Mediator from partially purified extracts [5]. Immediately adjacent to the RPB4/7 stalk region is the point from which the pol II CTD extends from the pol II enzyme (asterisk, Figure 5B). Because the pol II CTD is unstructured, it cannot be reliably localized within the Mediator–pol II–TFIIF cryo-EM map, nor has it been resolved from pol II crystal structure data. Based upon existing biochemical and biophysical studies, however, we propose the region highlighted green in Figure 5B represents the probable location of the pol II CTD within the PIC. This region corresponds to the site of pol II CTD–Mediator interaction identified previously using EM coupled with antibody labeling experiments with human Mediator bound to the pol II CTD [5]. Furthermore, this proposed pol II CTD location (Figure 5B) is proximal to the putative Cyclin H/CDK7 kinase module site within the human PIC (Figure 5A). Phosphorylation of the pol II CTD correlates with transcription initiation and elongation, and CDK7—a TFIIH subunit—is the major pol II CTD kinase within the PIC. The XPB/ERCC3 subunit within human TFIIH has been shown to interact with DNA elements just downstream of the transcription start site [14], which places TFIIH in an orientation such that its CDK7/Cyclin H lobe would be positioned near the proposed location of the pol II CTD in the Mediator–pol II–TFIIF structure. Although additional structural studies will be required to confirm the precise orientation of TFIIH within the human PIC, such TFIIH–pol II CTD co-localization also supports biochemical data that indicate a Mediator requirement for TFIIH-dependent pol II CTD phosphorylation within promoter-bound, human transcription complexes [9].

During transcription initiation, the newly transcribed RNA exits the pol II enzyme along the pol II RPB4/7 stalk [39],[40]. Within the Mediator–pol II–TFIIF assembly, the RPB4/7 stalk is oriented such that the nascent RNA could extend unobstructed from the PIC (Figure 5B). Thus, the architecture of the assembly ensures the transcript is readily accessible for capping enzymes and other RNA processing factors. Similarly, the pol II CTD emerges from the pol II enzyme at an exposed site adjacent to the RPB4/7 stalk (Figure 5B). In addition to binding Mediator within the PIC, the pol II CTD serves as an assembly platform for many RNA processing factors (e.g. capping, splicing, cleavage, and poly-adenylation factors) and is critical for generating stable, mature transcripts [41]. Based upon the Mediator–pol II–TFIIF assembly structure, RNA processing factors would have unhindered access to the pol II CTD.

Upon the transition from initiation to elongation, pol II must break contacts with the PIC. From a structural standpoint, it is currently unclear how pol II makes this transition; however, such a transition likely involves additional structural alterations within Mediator. Based upon the Mediator–pol II–TFIIF assembly structure, a portion of the Mediator head domain (1, Figure 5B) could pivot with a simple hinge-like motion back toward domain 2 (Figure 5B) to facilitate pol II promoter escape. In this way, Mediator could help regulate the pol II transition from initiation to elongation. Interestingly, activator-induced structural shifts within Mediator have been linked to activation of promoter-bound pol II complexes to a productively elongating state, indicating that activators likely contribute to this regulation [9]. Incorporation of additional PIC factors (e.g. TFIIE, TFIIH) might also trigger structural shifts in Mediator to facilitate pol II promoter escape. A conformational shift also occurs within pol II itself upon its transition to an elongating state and also when single-stranded DNA enters the active-site cleft [42]. These structural shifts may also disrupt pol II–Mediator contacts to favor promoter clearance and elongation. Further structural and functional studies will be required to better define how pol II–Mediator contacts are affected during the early stages of initiation.

**Figure 6:**
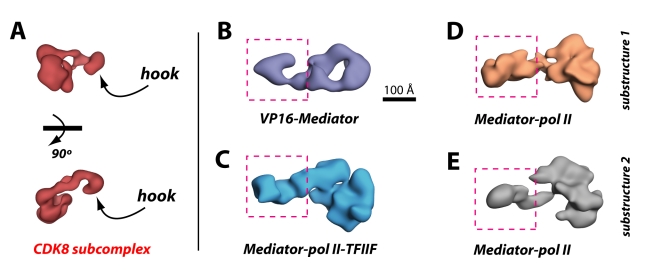
Pol II-induced structural rearrangements block potential Mediator-CDK8 subcomplex interactions. (A) Two views of the human CDK8 subcomplex [20]. (B) Structure of the human Mediator complex, bound to the activation domain of VP16 [19]. The CDK8 module hook domain binds the leg region (boxed area) of Mediator. Note the structural complementarity between the CDK8 submodule hook domain and the Mediator leg domain. Rearrangements in the leg region that ablate structural complementarity with the CDK8 submodule hook domain occur upon pol II binding in the presence (C) or absence (D,E) of TFIIF.

Perhaps most striking about the Mediator–pol II–TFIIF structure is that the majority of Mediator would remain exposed even upon assembly of the entire PIC (Figure 5A). Most of the surface area within the “body” of Mediator and all within the “leg” domain would remain accessible for potential protein-protein interactions. One well-established interaction involving the Mediator leg domain is with the 600 kDa CDK8 subcomplex, and biochemical and functional assays reveal the CDK8 subcomplex and pol II interact with Mediator in a mutually exclusive fashion [20],[32]. For example, the CDK8 subcomplex will not bind Mediator–pol II. The interface between the CDK8 subcomplex and the leg domain of Mediator is extensive and requires the Med13 subunit within the CDK8 subcomplex [20]. In fact, the CDK8 subcomplex contains a hook-like structural domain (Figure 6A) that interfaces with a complementary-shaped surface within the leg domain of VP16-Mediator (Figure 6B). This structural complementarity is abolished upon interaction with pol II, despite the fact that pol II binds the Mediator head domain over 100 Å from the leg domain–Med13 interface (Figure 6C). The pol II-induced structural shift within the leg domain is also observed without TFIIF—that is, even when pol II is not stably oriented within Mediator (Figure 6D and 6E). This structural shift in the leg domain likely occludes the Med13 interaction site, thereby regulating Mediator–CDK8 subcomplex interactions.

The mutually exclusive CDK8 subcomplex/pol II interactions with Mediator suggest a dynamic exchange at actively transcribing genes. Detachment of pol II from Mediator likely accompanies promoter clearance and transcription elongation and would allow subsequent CDK8 subcomplex–Mediator association [20]. This association would act to prevent a second pol II enzyme from immediately re-engaging the promoter. CDK8 subcomplex–Mediator association following pol II promoter clearance might also enable CDK8-Mediator to regulate transcription elongation. The human CDK8 subcomplex was recently identified as a regulator of transcription elongation for genes within the serum response network [43], and CDK8-Mediator appears to interact with elongation factors, including P-TEFb [32]. Potentially, pol II might remain in proximity to Mediator during elongation [44], which would allow a means by which CDK8-Mediator could simultaneously prevent re-initiation of transcription while affecting ongoing elongation events. Although further studies are required to explore this possibility, it is notable that ChIP data indicate Mediator occupancy within coding regions of active genes [45],[46], suggesting a juxtaposition of Mediator and pol II elongation complexes.

## Materials and Methods

### Isolation of Mediator–pol II–TFIIF or Mediator–pol II Complexes

VP16-Mediator and pol II were individually purified as endogenous complexes from HeLa nuclear extracts, whereas the two subunits of TFIIF, Rap74 and Rap30, were expressed recombinantly in *E. coli* and purified as described [20]. For each purification protocol (Mediator–pol II–TFIIF, Figure 2A, or Mediator–pol II, Figure 2C), the same Mediator and pol II samples were used. That is, a single, purified VP16-Mediator sample and a single, purified pol II sample were each split in half, with half of each sample used for the Mediator–pol II–TFIIF experiment and half for the Mediator–pol II experiment. The C-terminal domain (CTD) within the largest subunit of pol II, RPB1, can be extensively phosphorylated and this phosphorylation can negatively impact pol II association with Mediator [47]. Although the majority of the purified pol II sample appeared to be hypo-phosphorylated, we incubated pol II over a phosphatase resin (Sigma P0762) for 4 h at 4°C to ensure complete de-phosphorylation of the pol II CTD. This thoroughly de-phosphorylated pol II sample was used for the purifications outlined in Figure 2A and Figure 2C. The parallel Mediator–pol II and Mediator–pol II–TFIIF preparations were applied to separate glycerol step gradients. The gradient was designed to concentrate the full assemblies in the bottom fraction, while dispersing smaller complexes in earlier fractions. The gradient contained the following amounts of glycerol (in 20 mM HEPES, 100 µM EDTA, 150 mM KCl, 0.02% NP-40) from bottom to top: 100 µL 35%, 300 µL 30%, 800 µL 25%, and 800 µL 15%. The gradients were centrifuged at 55,000 rpm for 6 h at 4°C. The bottom (35%) glycerol gradient fraction was used for EM studies.

### Antibodies

Mediator was detected in Western blotting experiments using an antibody to Mediator subunit MED23 (Bethyl Cat. #A300-425A). Pol II was detected using an antibody to RPB1 (Santa Cruz sc-899), which detects both the hyper- and hypo-phosphorylated forms. TFIIF antibody (Rap74) was purchased from Austral Biologicals (Cat. #TM-101D-55).

### Mass Spectrometry

The purified VP16-Mediator complex (∼1 µg) and pol II complex (∼2 µg) fractions were precipitated at 4°C using 20% (v/v) TCA, 0.067 mg/mL insulin, and 0.067% (w/v) deoxycholate. Precipitated protein pellets were washed twice with −20°C acetone and air dried. Proteins were trypsin digested using a modified Filter-Aided Sample Prep (FASP) protocol [48]. Briefly, protein pellets were suspended with 4% (v/v) SDS, 0.1 M Tris pH 8.5, 10 mM TCEP, and incubated 30 min ambient to reduce disulfides. Reduced proteins were diluted with 8 M Urea, 0.1 M Tris pH 8.5. and iodoacetamide was added to 10 mM and incubated 30 min in total darkness. Reduced and alkylated proteins were then transferred to a Microcon YM-30 spin concentrator and washed twice with 8 M Urea, 0.1 M Tris pH 8.5 to remove SDS. Three washes with 2 M Urea, 0.1 M Tris pH 8.5 were performed, then trypsin and 2 mM CaCl_2_ was added and incubated approximately 2 h in a 37°C water bath. Digested peptides were eluted and acidified with 5% (v/v) formic acid.

Peptides were desalted online and fractionated with a Phenomenex Jupiter C18 (5 µm 300 Å; 0.25×150 mm) column using a two-dimensional LC/MS/MS method (Agilent 1100). Seven steps of increasing acetonitrile (3, 6, 9, 12, 16, 20, and 100% B; A: 20 mM ammonium formate pH 10, 4% acetonitrile; and B: 10 mM ammonium formate pH 10, 65% acetonitrile) at 5 µL/minute eluted peptides for a second dimension analysis on a Dionex Acclaim PepMap C18 (3 µm 100 Å; 0.075×150 mm) running a gradient at 0.2 µL/minute from 5% to 25% B in 100 min for steps one through six and 10% to 30% B in 100 min for step seven (A: 4% acetonitrile and B: 80% acetonitrile, both with 0.1% formic acid pH∼2.5). PepMap eluted peptides were detected with an Agilent MSD Trap XCT (3D ion trap) mass spectrometer.

All spectra were searched with Mascot v2.2 (Matrix Sciences) against the International Protein Index (IPI) database version 3.65 with two missed cleavages and mass tolerances of m/z ±2.0 Da for parent masses and ±0.8 Da for MS/MS fragment masses. Peptides were accepted above a Mascot ion score corresponding to a 1% false discovery rate (1% FDR) determined by a separate search of a reversed IPI v3.65 database. Peptides were then filtered and protein identifications were assembled using in-house software as described [49]. A listing of all polypeptides identified by MS is shown in Table S3 and Table S4.

### In Vitro Transcription

Reconstituted transcription reactions were completed on a DNA template with tandem GAL4 binding sites assembled into chromatin, as described [20].

### Negative Stain Electron Microscopy

Sample (either Mediator–pol II–TFIIF or Mediator–pol II) was applied to a glow-discharged carbon-coated copper EM grid (EMS cat. #CF400-Cu) and washed twice with 5% trehalose buffer (20 mM HEPES, pH 7.9, 0.1 mM EDTA, and 100 mM KCl). Grids were floated on a droplet of water and then stained with 2% uranyl acetate in water. Images were recorded on Kodak SO-163 film using a Tecnai F20 microscope operated at 200 kV. Untilted (0°) and tilted (25°–45°) specimen images were collected at 29,000× magnification with a defocus range of −1.0 to −3.5 µm. The film was digitized with a sample-scale pixel size of 4.29 angstroms. Individual particle images were windowed into 161×161 pixel boxes using the Web interface of the SPIDER image processing software [50]. The untilted images were subjected to unsupervised (reference-free) 2D classification based upon a k-means clustering algorithm [25].

For the Mediator-pol II-TFIIF data set, a total of 8,923 tilted and untilted pairs were selected. Each 2D class—derived from the untilted data set—contained dozens to hundreds of individual single-particle images. Particles grouped within the same class represented complexes with a similar orientation on the EM grid; particles in different 2D classes represented an alternate orientation of the assembly (e.g. “side” or “top” views) or potentially might reflect an alternate conformational state. The corresponding tilted images within each 2D class were back-projected to generate 3D model structures, which were then cross-correlated and subjected to hierarchical clustering using the statistical program package R [51]. One branch of the cluster dendrogram contained well-correlating volumes with images that could be combined into a single converging reference volume, and these data served as the negative stain data set. The tilted and untilted data were used for iterative 2D projection matching to refine the structure.

The initial Mediator–pol II–TFIIF reference volume was generated from 3,215 tilted images. This reference was then refined with 6,430 total images (tilted + untilted) using projection matching with angular steps of 15°, 10°, and 5°. The final refinement step included 5,047 (78%) of these images. All half-volumes were individually masked to 2 MDa (to generate non-identical masks) prior to resolution assessment using the 0.5 Fourier shell criterion [52].

The Mediator–pol II reference volume was generated from 3,134 tilted images. This reference was then refined with 6,268 total images (tilted + untilted). The resolution of this structure improved more slowly, so angular steps of 15°, 10°, 5°, 4°, 3°, and 3° were used. The final refinement step for this volume included 4,864 (78%) of these images and the 0.5 Fourier shell criterion was applied to determine the resolution [52].

Because an excess of VP16-Mediator was included in the sample preparation, we anticipated some free VP16-Mediator would be present within each data set. To deal with this heterogeneity, efforts were taken to generate negative stain structures of Mediator–pol II–TFIIF and Mediator–pol II free from potential contamination with unbound VP16-Mediator. Images of free VP16-Mediator were removed using a single round of projection matching (15° angular step) with two input reference volumes—the negative stain volume described above and a previously determined structure of free VP16-Mediator [19]. Particle images with a higher correlation to free VP16-Mediator were removed from the data set, and both the Mediator–pol II–TFIIF and Mediator–pol II structures were refined again. This refinement was identical to that described above, except that only untilted images were used for projection matching. The Mediator–pol II–TFIIF structure was refined using 15°, 10°, 5°, and 5° steps with 2,390 (74%) images included in the final volume. The Mediator–pol II structure was refined using 15°, 10°, 5°, 5°, and 4° steps with 2,223 (71%) images included in the final volume.

### Cryo-Electron Microscopy

Cryo-negative stain samples for electron microscopy were prepared largely as described [53]. Purified complexes were added to a glow-discharged thin carbon-coated holey carbon copper mesh grid (EMS cat. #CF424-50). A buffer containing 5% trehalose, 20 mM HEPES, 100 mM KCl, and 100 µM EDTA was used to wash the grid 3 times to remove excess glycerol. The sample grid was then floated on a drop of stain (1.2 M ammonium molybdate pH 7.5). Excess stain was blotted away and the grid was plunge frozen in liquid ethane to vitrify the sample.

Imaging was carried out at liquid nitrogen temperatures under low-dose conditions on a Tecnai F20 FEG microscope (200 kV). Images were recorded at 29,000× magnification on Kodak SO-163 film with a defocus range of −1.0 to −4.5 µm. Negatives were then digitized to 4.29 angstroms per pixel using a Microtek Scanmaker i900 for Mediator-RNA pol II-TFIIF and 4.22 angstroms per pixel using a Nikon Super Coolscan 9000 ED for Mediator–pol II. Micrographs were screened for astigmatism and drift using ctfit (within the EMAN [54] software package) by removing those with distorted or poorly defined Thon rings in the power spectrum. Individual particle images were manually selected from high-quality micrographs before being windowed into 161×161 pixel boxes for further processing.

The contrast transfer function parameters were estimated for each micrograph using the program CTFFIND3 [55]. Using these parameters, individual images were CTF-corrected for phase on a per-micrograph basis. The cryo-EM data set for the Mediator–pol II–TFIIF assembly included 10,856 images. The cryo-EM data set for the Mediator–pol II assembly included 7,962 images.

The appropriate negative stain structures or cryo-EM structures (generated by three-dimensional variance and sub-classification of images—see below), Butterworth filtered to 57 Å, served as initial references for multi-reference projection matching refinement [56],[57]. Multi-reference refinements were completed as described [57]. The resolution of each reconstruction was calculated using the 0.5 Fourier shell criterion [52] or the 3σ-threshold criterion [58]. For Mediator–pol II–TFIIF, using the 0.5 Fourier shell criterion, the resolution was found to be 36 Å, whereas using the 3σ-threshold criterion indicated 26 Å resolution. For Mediator–pol II substructure 1, the 0.5 Fourier shell and 3σ criteria specified a resolution of 34 Å and 29 Å, respectively. For Mediator–pol II substructure 2, the 0.5 Fourier shell and 3σ criteria indicated 36 Å and 31 Å resolution, respectively. The reconstructions were displayed after filtering to the values obtained using the 0.5 Fourier shell criterion.

In order to estimate the degree of structural homogeneity in the cryo-EM Mediator–pol II–TFIIF data set, several multi-reference refinements were completed using different percentages of the data. Volumes were created using 46%, 55%, 62%, or 78% of the 10,856 cryo-EM images, based upon their cross-correlation coefficient. The structure generated using 78% of the data converged poorly and resulted in a highly discontinuous structure. The assembly structures generated using 46%, 55%, or 62% of the data converged to very similar solutions that cross-correlated in the 0.94–0.98 range. Despite containing additional data, the 55% and 62% reconstructions did not result in an improved resolution, and trace discontinuities in the volumes appeared at the 1.8 MDa threshold. Consequently, the assembly reconstruction produced using 46% of the data set was used for crystal structure docking and projection matching experiments. Similar refinement trials were used to determine that 59% of the data would be included in the final 3D reconstruction of the Mediator–pol II binary complex.

### Three-Dimensional Variance and Sub-Classification

To further probe for alternate structural states within the Mediator–pol II–TFIIF assembly and to potentially separate out structurally distinct conformers, we carried out a 3D variance analysis, developed by Penczek and co-workers [24]. This methodology identifies regions of structural variability by comparing the variance within the structure pixel-by-pixel, relative to background. Focused classification within a region of high variance has the potential to segregate structurally distinct assemblies, especially in cases in which the variance is well-localized and there are a few clearly distinguishable conformational states. Importantly, this technique has proven effective in identifying structural flexibility within a number of multi-subunit complexes, including human transcription complexes [22],[29],[57]. Implementation of this approach to the Mediator–pol II–TFIIF data (i.e. data that partitioned to the Mediator–pol II–TFIIF structure during the multi-reference refinement), however, did not improve the resolution of the structure overall. In fact, this approach—which will partition the Mediator–pol II–TFIIF data to generate two substructures—resulted in two virtually identical structures with consistent pol II docking orientations. This is likely indicative of flexibility within the assembly and suggests a relatively stable Mediator–pol II–TFIIF conformational state about which the structure oscillates. Taken together with the *de novo* cryo-EM reconstruction results described above, these results indicate that although the structure shown in Figure 3A is stable and represents a major entity within the Mediator–pol II–TFIIF cryo-EM data set, the flexible nature of the assembly precludes further improvement of spatial resolution.

The 3D variance within the refined Mediator–pol II–TFIIF or Mediator–pol II cryo-EM structures was estimated by creating and comparing 500 re-sampled volumes using a bootstrap technique as described [24]. Focused classification within the area of the 3D reconstruction displaying the highest variance was then used to separate the data into more homogenous groups [22]. Specifically, the refined structure was projected in 28 directions (angular interval of 25 degrees) and each image used in the final round of angular refinement was matched to the highest correlating projection. This classification step produced 28 groups of images, each representing a similar orientation of the assembly, or “projection groups.” The highest peak in the 3D variance density map was then used to generate a mask for classification by projection in each of the directions corresponding to the 28 groups of images. For each projection group, k-means classification within the mask was used to separate the images into two subclasses, which in turn were sorted by visual inspection into one of two groups. Group 1 contained subclasses with less apparent density, and group 2 contained subclasses with more apparent density. The original structure was then subjected to one round of refinement using each group of images to generate two new reference structures. Finally, multi-reference projection matching was used to refine these substructures.

### Assessment of Potential Model Bias

Whereas the generation of reference-free classes that closely resembled reprojections of either Mediator–pol II–TFIIF or free VP16-Mediator indicated that both entities were present within the cryo-EM data set (Figure S4), we wanted to further confirm that each structure (Figure 3 and Figure S3) did not result from model bias [26] during angular refinement. To do this, reciprocal refinements were completed for Mediator–pol II–TFIIF and free VP16-Mediator. In one case, the free VP16-Mediator reference was refined using the data that had previously been partitioned into the Mediator–pol II–TFIIF data set. As expected, pol II density was built into the structure and the general shape of the Mediator–pol II–TFIIF assembly began to emerge during angular refinement. For example, the cleft between lobes 1 and 2 and the cleft between lobe 3 and the body/leg of Mediator became re-defined in the structure (Figure S7 and unpublished data). This suggested that structural features observed for Mediator–pol II–TFIIF do not result from initial model bias. In the second case, the data representing free VP16-Mediator was used to refine an initial Mediator–pol II–TFIIF reference structure. Again, as expected, density representing pol II disappeared from the Mediator head region and the pocket domain located between lobes 1, 2, and 3 of Mediator emerged during the refinement (Figure S7 and unpublished data). Because key structural features of the Mediator–pol II–TFIIF and free VP16-Mediator models were preserved even when a reference volume lacking these features was used as a starting point for refinement, model bias did not negatively impact the cryo-EM reconstructions of either the Mediator–pol II–TFIIF assembly or the free VP16-Mediator structure.

### Pol II Crystal Structure Docking

A yeast crystal structure of pol II (PDB 1Y1V) [28], with the TFIIS fragment removed, was roughly fit into the desired EM structure using Chimera [59]. This visual docking was refined using the FFT-Accelerated 6D Exhaustive Search program of Situs [27]. Using the search tool Colores [60], an exhaustive search of translational and rotational space was performed and as expected we did not see any dependence of the best docking fit upon the initial position. Because the 12-subunit yeast pol II structure 1Y1V was shown previously to fit well into the human pol II EM structure [29], the 1Y1V structure was chosen to be displayed. Docking was also completed using each of the complete 12-subunit pol II structures found in the RCSB Protein Data Bank (1NT9, 1PQV, 1WCM, 1Y1W, 1Y1Y, 1Y77, 2B8K, 2B63, 2JA5, 2JA6, 2JA7, 2JA8, 2VUM, 3FKI, 3HOU, 3HOV, 3HOW, 3HOX, 3HOY, 3HOZ, 3K1F), with identical results.

### Pol II Projection Matching

The orientation of pol II was also determined using 2D projection matching [10] of projection averages of each cryo-EM structure (Mediator–pol II–TFIIF or Mediator–pol II) and re-projections generated from a previously published cryo-negative stain human pol II 3D model [29]. The refined 3D reconstruction (Mediator–pol II–TFIIF or Mediator–pol II) was projected into 84 directions (15°intervals) and each image included in the reconstruction was matched to the best-correlating projection. Images from each projection group were aligned in 2D and averaged to yield a “view average.” Because many projections of the Mediator–pol II–TFIIF or Mediator–pol II assembly structures contain overlapping Mediator and pol II densities, density contributions from pol II alone often could not be distinguished. To ensure the most accurate comparison of pol II features, the view average corresponding to the projection of the assembly that had the least amount of Mediator density overlapping with pol II density was used for this analysis. Re-projections of the human pol II structure were generated using a 5° angular step for a total of 1,596 projections. Each of these projections was matched to the area of the assembly 2D projection average containing pol II (as determined by Situs docking).

A plot was generated to visualize areas of best correlation for a single projection average. The axes of the plot are the phi and theta angles. Larger points indicate higher correlation between a particular 2D re-projection of pol II and the projection average of the assembly structure. For ease of viewing the peak correlations, the radius of each point is proportional to 10 raised to the studentized residual (10 ^(value - mean)/standard deviation^). Each cluster of large points indicates a well-correlating orientation of pol II. The highest correlation was observed with an orientation of pol II that closely matched the 3D docking result (Figure S6).

## Supporting Information

Figure S1 Negative stain EM analysis of the Mediator–pol II–TFIIF assembly. (A) Negative stain micrograph of the Mediator–pol II–TFIIF assembly. Scale bar: 100 nm. (B) Several views of the random conical tilt, negative stain EM reconstruction of the Mediator–pol II–TFIIF assembly. The structure was refined to 42 Å resolution and is shown rendered to 1.8 MDa. (C) Angular distribution of images included in the Mediator–pol II–TFIIF negative stain reconstruction. (D) Resolution curve for the negative stain reconstruction of the Mediator–pol II–TFIIF assembly. The Fourier shell correlation is shown in blue and the 3σ noise curve is shown in red. (E) Comparison of projections of the Mediator–pol II–TFIIF assembly and projections of VP16-Mediator only. Extra density resulting from pol II binding is observed in the head region of Mediator.
(2.39 MB EPS)

Figure S2 Additional information and statistics pertaining to the Mediator–pol II–TFIIF cryo-EM reconstruction. (A) Cryo-negative stain micrograph of the Mediator–pol II–TFIIF assembly. Scale bar: 100 nm. (B) Power spectrum of the micrograph shown in part A. Micrographs included in the reconstruction display clear Thon rings indicating a minimum of drift and astigmatism. (C) Resolution curve for the cryo-EM reconstruction of the Mediator–pol II–TFIIF assembly. The Fourier shell correlation is shown in blue and the 3σ noise curve is shown in red. (D) Angular distribution of images included in the Mediator–pol II–TFIIF cryo-EM reconstruction.
(6.16 MB EPS)

Figure S3 The refined VP16-Mediator structure obtained from the Mediator–pol II–TFIIF cryo-EM data set resembles the previously published VP16-Mediator structure obtained from negatively stained samples. (A) Three views of the previously published negative stain VP16-Mediator reconstruction [19]. (B) Three corresponding views of the cryo-EM VP16-Mediator reconstruction. Free VP16-Mediator complexes were present within the Mediator–pol II–TFIIF data set; these particles were used to generate an independent 3D reconstruction of VP16-Mediator from cryo-EM data. (C) Resolution curve for the cryo-EM reconstruction of VP16-Mediator. The Fourier shell correlation is shown in blue and the 3σ noise curve is shown in red. (D) Angular distribution of images included in the VP16-Mediator cryo-EM reconstruction.
(1.82 MB EPS)

Figure S4 Reference-free 2D classes generated from the cryo-EM Mediator–pol II–TFIIF data set resemble projections and views generated from the Mediator–pol II–TFIIF assembly reconstruction. (A) Representative 2D classes or projections that correspond to the Mediator–pol II–TFIIF assembly. (B) Representative 2D classes or projections that correspond to the free VP16-Mediator structure, which was also present within the cryo-EM data set. The similarity of the reference-free classes to the projections and classes generated using the Mediator–pol II–TFIIF reconstruction (in A) or the VP16-Mediator reconstruction (in B) demonstrates that potential bias from the negative stain reference volumes was minimal. Thresholded proj. refers to 2D projections generated from the refined, Mediator–pol II–TFIIF 3D reconstruction, masked to a 2.0 MDa threshold; proj. represents 2D projections generated from the refined, Mediator–pol II–TFIIF 3D reconstruction that were not thresholded. View avg refers to 2D class averages generated from actual cryo-EM data in which 2D projections of the refined assembly guide classification (i.e. supervised classification); ref-free class avg. represent 2D classes that resulted from k-means classification (no reference used) of the entire cryo-EM data set.
(2.13 MB EPS)

Figure S5 Structural features of human pol II at 36 Å resolution. Shown are the yeast pol II crystal structure alongside the human pol II complex rendered at 36 Å resolution. Note that at this resolution, major pol II structural features such as the cleft, jaws, and stalk are clearly defined.
(1.65 MB EPS)

Figure S6 Pol II projection matching experiments corroborate the pol II docking result for the Mediator–pol II–TFIIF assembly. (A) Plot of the correlations of projections of pol II and a view average of Mediator–pol II–TFIIF. The phi and theta angles of the pol II projections are plotted on the *y*- and *x*-axes, respectively. The radius of each point is proportional to the correlation coefficient for that particular pol II orientation (see Materials and Methods). The circled cluster of points indicates the best-correlating orientations of pol II, and the point denoted by the arrow represents the highest correlating pol II projection. (B) The projection of Mediator–pol II–TFIIF and view average used for projection matching (panel 1 and 2); panel 3 shows the view average with the area containing pol II highlighted (bright circle). The region within this circle was used for pol II projection matching. Panel 4 shows the best-correlating pol II projection, which corresponds to the arrow in (A). (C) A comparison of the pol II docking result and the orientation determined from projection matching. Panel 1 shows the docking fit from Situs [27] with pol II displayed in red ribbon and Mediator–pol II–TFIIF in blue mesh. Panel 2 shows the projection matching best fit, with pol II in light blue and Mediator–pol II–TFIIF in blue mesh. Panel 3 shows the pol II fit alone, and panel 4 shows the same pol II orientation shown with the “solid” viewing option within Chimera [59]. This viewing option allows interior features to be more apparent and approximates an actual 2D projection view. (D) The fits from part C shown from an alternate angle—the “front” view of the Mediator–pol II–TFIIF assembly. Panel 1 shows both the pol II docking fit in red and the pol II projection matching fit in light blue. The Mediator–pol II–TFIIF density is shown in blue mesh. Panel 2 shows both pol II fits overlaid, without the Mediator–pol II–TFIIF cryo-EM density. Panel 3 shows the docking fit only, whereas panel 4 shows the projection matching fit only. Note the similarity of the pol II orientation resulting from either the docking or the projection matching (compare panel 3 and panel 4).
(1.65 MB EPS)

Figure S7 Model illustrating Mediator–pol II–TFIIF binding and Mediator structural shifts induced by pol II–TFIIF binding. Different views of the free VP16-Mediator structure [19] are shown in purple; pol II is shown in red ribbon (PDB 1Y1V); the Mediator–pol II–TFIIF structure is shown in blue. The orientation and direction from which pol II interfaces with Mediator is based upon the Mediator–pol II–TFIIF cryo-EM structure shown in Figure 3A. The “side 1” view highlights three Mediator domains (labeled 1, 2, 3) and their probable locations following pol II–TFIIF binding. The Mediator head, body, and leg domains are labeled alongside the “top” view of VP16-Mediator. Note that structural shifts occur within the head, body, and leg domains of Mediator upon pol II–TFIIF binding. Dashed lines indicate pol II approach from behind the plane. *The RPB4/7 stalk is directed away from the viewer; **the RPB4/7 stalk is pointed toward the viewer.
(2.49 MB EPS)

Figure S8 Image processing information pertaining to the Mediator–pol II cryo-EM reconstructions. (A) Cryo-negative stain micrograph of the Mediator–pol II assembly. Scale bar: 100 nm. (B) Resolution curve for the cryo-EM reconstruction of Mediator–pol II, substructure 1. The Fourier shell correlation is shown in blue and the 3σ noise curve is shown in red. (C) Angular distribution of images included in the cryo-EM reconstruction of Mediator–pol II, substructure 1. (D) Resolution curve for the cryo-EM reconstruction of Mediator–pol II, substructure 2. The Fourier shell correlation is shown in blue and the 3σ noise curve is shown in red. (E) Angular distribution of images included in the cryo-EM reconstruction of Mediator–pol II, substructure 2.
(4.39 MB EPS)

Figure S9 3D variance within the Mediator–pol II cryo-EM data. The peak areas of 3D variance are shown in blue, superimposed on each of the two Mediator–pol II substructures. Single-particle images corresponding to either substructure 1 or substructure 2 were classified using the 3D variance at region 1. Note that 3D variance region 1 is exposed within substructure 1 but occupied by protein density within substructure 2. The Mediator–pol II substructure 1 is shown in peach and substructure 2 is shown in gray.
(2.16 MB EPS)

Figure S10 The human PIC model and comparison with yeast PIC models. (A) Human PIC model based upon this study. In contrast to the yeast PIC models, the human PIC model contains an open, complementary surface for TFIID incorporation into the assembly; upstream and downstream DNA is accessible for potential interactions with TFIID and other transcription factors; the TFIIH assembly site, downstream from the transcription start site, is accessible and positions TFIIH adjacent to the head domain of human Mediator. Current yeast PIC models are based upon EM data of yeast Mediator and a 10-subunit yeast pol II enzyme [10], or EM data of a 7-subunit yeast Mediator head module with 2 subunits (RPB4/7) of yeast pol II [11]. Note that pol II orientation is not consistent in these yeast PIC models (e.g. compare Figure 3, [13], and Figure 5, [11]): RPB4/7 orient toward the Mediator tail domain in one model [10],[13], whereas RPB4/7 are proposed to interact with the Mediator head domain in another model [11]. Neither yeast PIC model appears to be compatible with TFIID binding—the TFIID interaction surface (adjacent the RPB 3/10/11/12 subunits) is blocked by Mediator density in these models. Upstream DNA—important for TFIID interactions and transcription factor binding—is also blocked by Mediator density in these models. The location of TFIIH in each yeast PIC model would allow for interaction with downstream DNA; however, the location of TFIIH in each yeast PIC model does not appear consistent with biochemical and biophysical data that indicate yeast TFIIH interacts directly with the Mediator head module subunit MED11 [61]. (B) Rough outline of EM structures of human TFIID [36] and TFIIH [35], shown at approximately the same relative scale as the human PIC model in (A).
(7.97 MB EPS)

Figure S11 MS/MS spectra supporting the presence of MED28 within the VP16-Mediator sample. Shown are representative MS/MS spectra that identify MED28; MED28 represented the only Mediator subunit not identified following 1% FDR analysis (Table 1). Potentially, MED28 could dissociate from Mediator during the purification procedure (Figure 1C). This does not appear to be the case, as MED28 peptides appear to be represented, although not at 99% confidence.
(1.01 MB EPS)

Table S1 List of PDB files used for pol II docking calculations. Note that indistinguishable docking results were calculated for each 12-subunit pol II structure PDB file. The PDB 1Y1V structure is shown throughout the article (with the TFIIS density removed) because this structure was found to correspond most closely to the human pol II cryo-EM structure [29].
(0.04 MB DOC)

Table S2 Sequence identity of human and yeast Mediator subunits.
(0.07 MB DOC)

Table S3 Summary of all proteins identified from mass spectrometry analysis of VP16-Mediator. Sample isolated as shown in Figure 1C; glycerol gradient fractions 13–15 were analyzed. Spectral counts corresponding to keratin were removed from this list.
(0.23 MB DOC)

Table S4 Summary of all proteins identified from mass spectrometry analysis of pol II. Sample isolated as shown in Figure 1B. Note that for structural and functional work completed here, a glycerol gradient sedimentation step was completed in order to isolate the Mediator–pol II or Mediator–pol II–TFIIF assemblies. The pol II sample analyzed using mass spectrometry was evaluated prior to glycerol gradient sedimentation, and thus sub-stoichiometric contaminants are present in this analysis. Nonetheless, it is evident from the spectral counts as well as the pol II silver-stained gel (Figure 1B) that pol II is predominant in this sample even prior to the glycerol gradient purification step. Spectral counts corresponding to keratin were removed from this list.
(0.46 MB DOC)

Movie S1 Structure of Mediator–pol II–TFIIF. A 3D surface representation of the cryo-EM reconstruction of Mediator–pol II–TFIIF, rendered to 1.8 MDa, is shown in solid blue. The structure is rotated 360 degrees about the horizontal axis, followed by a rotation of 360 degrees about the vertical axis. The Mediator–pol II–TFIIF surface is then displayed as blue mesh with a pol II crystal structure (PDB 1Y1V) shown in red ribbon in its docked orientation. The cryo-EM structure and docked pol II are then rotated about the horizontal and vertical axis as before, with the rotation paused to highlight views of the complex shown in Figure 3. Scale bar represents 100 Å.
(10.00 MB MOV)
