# Supplementary material for: Molecular Architecture of the Human Mediator–RNA Polymerase II–TFIIF Assembly
Source: PLoS Biol. 2011 Mar 29;9(3):e1000603. doi: 10.1371/journal.pbio.1000603 (PMC3066130; doi:10.1371/journal.pbio.1000603)
Supplement: Table S1 — List of PDB files used for pol II docking calculations. Note that indistinguishable docking results were calculated for each 12-subunit pol II structure PDB file. The PDB 1Y1V structure is shown throughout the article (with the TFIIS density removed) because this structure was found to correspond most closely to the human pol II cryo-EM structure [29]. (0.04 MB DOC) [file pbio.1000603.s012.doc]

| **PDB ID** | **Date** | **Resolution (Å)** | **Description** |
| --- | --- | --- | --- |
| 1NT9 | 2003 | 4.20 | Complete 12-subunit RNA polymerase II |
| 1PQV | 2003 | 3.80 | RNA polymerase II-TFIIS complex |
| 1Y1V | 2004 | 3.80 | Refined RNA Polymerase II-TFIIS complex |
| 1Y1W | 2004 | 4.00 | Complete RNA Polymerase II elongation complex |
| 1Y1Y | 2004 | 4.00 | RNA Polymerase II-TFIIS-DNA/RNA complex |
| 1Y77 | 2004 | 4.50 | Complete RNA Polymerase II elongation complex with substrate analogue GMPCPP |
| 1WCM | 2005 | 3.80 | Complete 12-Subunit RNA Polymerase II at 3.8 Ang |
| 2B63 | 2006 | 3.80 | Complete RNA Polymerase II-RNA inhibitor complex |
| 2B8K | 2006 | 4.15 | 12-subunit RNA Polymerase II |
| 2JA5 | 2007 | 3.80 | CPD Lesion Containing RNA Polymerase II Elongation Complex A |
| 2JA6 | 2007 | 4.00 | CPD Lesion Containing RNA Polymerase II Elongation Complex B |
| 2JA7 | 2007 | 3.80 | CPD Lesion Containing RNA Polymerase II Elongation Complex C |
| 2JA8 | 2007 | 3.80 | CPD Lesion Containing RNA Polymerase II Elongation Complex D |
| 2VUM | 2008 | 3.40 | Alpha-Amanitin Inhibited Complete RNA Polymerase II Elongation Complex |
| 3FKI | 2009 | 3.88 | 12-Subunit RNA Polymerase II Refined with Zn-SAD data |
| 3HOU | 2009 | 3.20 | Complete RNA polymerase II elongation complex I with a T-U mismatch |
| 3HOV | 2009 | 3.50 | Complete RNA polymerase II elongation complex II |
| 3HOW | 2009 | 3.60 | Complete RNA polymerase II elongation complex III with a T-U mismatch and a frayed RNA 3'-uridine |
| 3HOX | 2009 | 3.65 | Complete RNA polymerase II elongation complex V |
| 3HOY | 2009 | 3.40 | Complete RNA polymerase II elongation complex VI |
| 3HOZ | 2009 | 3.65 | Complete RNA polymerase II elongation complex IV with a T-U mismatch and a frayed RNA 3'-guanine |
| 3K1F | 2009 | 4.30 | Crystal structure of RNA Polymerase II in complex with TFIIB |
