# Supplementary material for: Molecular Architecture of the Human Mediator–RNA Polymerase II–TFIIF Assembly
Source: PLoS Biol. 2011 Mar 29;9(3):e1000603. doi: 10.1371/journal.pbio.1000603 (PMC3066130; doi:10.1371/journal.pbio.1000603)
Supplement: Table S2 — Sequence identity of human and yeast Mediator subunits. (0.07 MB DOC) [file pbio.1000603.s013.doc]

| Yeast | % Identity | Human |
| --- | --- | --- |
| MED1 | 7.2% | MED1 |
| MED2 |  |  |
| MED3 |  |  |
| MED4 | 20.7% | MED4 |
| MED5 |  |  |
| MED6 | 18.4% | MED6 |
| MED7 | 23.2% | MED7 |
| MED8 | 9.1 - 15.2% | MED8 |
| MED9 | 16.4% | MED9 |
| MED10 | 24.4% | MED10 |
| MED11 | 17.7% | MED11 |
| MED14 | 13.5% | MED14 |
| MED15 | 18.5 - 19.1% | MED15 |
| MED16 | 14.8% | MED16 |
| MED17 | 13.5% | MED17 |
| MED18 | 11.5% | MED18 |
| MED19 | 9.4% | MED19 |
| MED20 | 10.5% | MED20 |
| MED21 | 25.0% | MED21 |
| MED22 | 11.3% | MED22 |
|  |  | MED23 |
|  |  | MED24 |
|  |  | MED25 |
|  |  | MED26 |
|  |  | MED27 |
|  |  | MED28 |
|  |  | MED29 |
|  |  | MED30 |
| MED31 | 27.5% | MED31 |
|  |  |  |
| **% Identity** |  |  |
| No ortholog |  |  |
| 0-10% |  |  |
| 10 to 20% |  |  |
| 20-30% |  |  |
