# Supplementary material for: Molecular Architecture of the Human Mediator–RNA Polymerase II–TFIIF Assembly
Source: PLoS Biol. 2011 Mar 29;9(3):e1000603. doi: 10.1371/journal.pbio.1000603 (PMC3066130; doi:10.1371/journal.pbio.1000603)
Supplement: Table S3 — Summary of all proteins identified from mass spectrometry analysis of VP16-Mediator. Sample isolated as shown in Figure 1C; glycerol gradient fractions 13–15 were analyzed. Spectral counts corresponding to keratin were removed from this list. (0.23 MB DOC) [file pbio.1000603.s014.doc]

| **Spectral Counts** | Gene | Prot MW | Protein descriptor | |  |  |  |  |  |  |  |  |  |
| --- | --- | --- | --- | --- | --- | --- | --- | --- | --- | --- | --- | --- | --- |
| 184 | MED1 | 168478 | ISOFORM 1 OF MEDIATOR OF RNA POLYMERASE II TRANSCRIPTION SUBUNIT 1."" | | | | | | |  |  |  |  |
| 175 | MED15 | 86753 | ISOFORM 1 OF MEDIATOR OF RNA POLYMERASE II TRANSCRIPTION SUBUNIT 15."" | | | | | | |  |  |  |  |
| 118 | MED14 | 160607 | MEDIATOR OF RNA POLYMERASE II TRANSCRIPTION SUBUNIT 14."" | | | | | |  |  |  |  |  |
| 83 | MED16 | 96793 | ISOFORM 1 OF MEDIATOR OF RNA POLYMERASE II TRANSCRIPTION SUBUNIT 16."" | | | | | | |  |  |  |  |
| 79 | MED26 | 65446 | ISOFORM 1 OF MEDIATOR OF RNA POLYMERASE II TRANSCRIPTION SUBUNIT 26."" | | | | | | |  |  |  |  |
| 67 | POLR2A | 217206 | DNA-DIRECTED RNA POLYMERASE II SUBUNIT RPB1."" | | | | |  |  |  |  |  |  |
| 57 | MED31 | 15805 | MEDIATOR OF RNA POLYMERASE II TRANSCRIPTION SUBUNIT 31."" | | | | | |  |  |  |  |  |
| 52 | MED24 | 110305 | MEDIATOR OF RNA POLYMERASE II TRANSCRIPTION SUBUNIT 24."" | | | | | |  |  |  |  |  |
| 52 | MED20 | 23222 | MEDIATOR OF RNA POLYMERASE II TRANSCRIPTION SUBUNIT 20."" | | | | | |  |  |  |  |  |
| 51 | MED24 |  |  |  |  |  |  |  |  |  |  |  |  |
| 49 | SNRNP200 | 244508 | ISOFORM 1 OF U5 SMALL NUCLEAR RIBONUCLEOPROTEIN 200 KDA HELICASE."" | | | | | |  |  |  |  |  |
| 46 | MED17 | 72876 | ISOFORM 1 OF MEDIATOR OF RNA POLYMERASE II TRANSCRIPTION SUBUNIT 17."" | | | | | | |  |  |  |  |
| 43 | MED4 | 29745 | MEDIATOR OF RNA POLYMERASE II TRANSCRIPTION SUBUNIT 4."" | | | | |  |  |  |  |  |  |
| 39 | MED23 |  |  |  |  |  |  |  |  |  |  |  |  |
| 35 | MAGED2 | 64954 | ISOFORM 1 OF MELANOMA-ASSOCIATED ANTIGEN D2."" | | | | |  |  |  |  |  |  |
| 31 | MED25 | 84389 | ISOFORM 4 OF MEDIATOR OF RNA POLYMERASE II TRANSCRIPTION SUBUNIT 25."" | | | | | | |  |  |  |  |
| 29 | EFTUD2 | 109436 | 116 KDA U5 SMALL NUCLEAR RIBONUCLEOPROTEIN COMPONENT."" | | | | | |  |  |  |  |  |
| 26 | MED9 | 16403 | MEDIATOR OF RNA POLYMERASE II TRANSCRIPTION SUBUNIT 9."" | | | | |  |  |  |  |  |  |
| 25 | MED10 | 15688 | MEDIATOR OF RNA POLYMERASE II TRANSCRIPTION SUBUNIT 10."" | | | | | |  |  |  |  |  |
| 24 | RUVBL2 | 51157 | RUVB-LIKE 2."" | |  |  |  |  |  |  |  |  |  |
| 24 | MED29 | 23473 | INTERSEX-LIKE."" | |  |  |  |  |  |  |  |  |  |
| 24 | MED18 | 24453 | CDNA FLJ51219. HIGHLY SIMILAR TO HOMO SAPIENS MEDIATOR OF RNAPOLYMERASE II TRANSCRIPTION. SUBUNIT 18 HOMOLOG (MED18). MRNA."" | | | | | | | | | | |
| 20 | MED22 | 22221 | ISOFORM SURF5B OF MEDIATOR OF RNA POLYMERASE II TRANSCRIPTION SUBUNIT22."" | | | | | | |  |  |  |  |
| 19 | MED8 | 32819 | ISOFORM 2 OF MEDIATOR OF RNA POLYMERASE II TRANSCRIPTION SUBUNIT 8."" | | | | | | |  |  |  |  |
| 19 | MED7 | 27245 | MEDIATOR OF RNA POLYMERASE II TRANSCRIPTION SUBUNIT 7."" | | | | |  |  |  |  |  |  |
| 19 | MED30 | 20277 | MEDIATOR OF RNA POLYMERASE II TRANSCRIPTION SUBUNIT 30."" | | | | | |  |  |  |  |  |
| 17 | MRPS22 | 41280 | 28S RIBOSOMAL PROTEIN S22. MITOCHONDRIAL."" | | | |  |  |  |  |  |  |  |
| 17 | MED27 | 35432 | ISOFORM 1 OF MEDIATOR OF RNA POLYMERASE II TRANSCRIPTION SUBUNIT 27."" | | | | | | |  |  |  |  |
| 16 | PRPF8 | 273600 | PRE-MRNA-PROCESSING-SPLICING FACTOR 8."" | | | |  |  |  |  |  |  |  |
| 15 | POLR2E | 24551 | DNA-DIRECTED RNA POLYMERASES I. II. AND III SUBUNIT RPABC1."" | | | | | |  |  |  |  |  |
| 15 | MED6 | 29298 | CDNA FLJ52756. HIGHLY SIMILAR TO RNA POLYMERASE TRANSCRIPTIONALREGULATION MEDIATOR. SUBUNIT 6 HOMOLOG."" | | | | | | | | | |  |
| 15 | PRPF6 | 106925 | PRE-MRNA-PROCESSING FACTOR 6."" | | |  |  |  |  |  |  |  |  |
| 14 | POLR2B | 133897 | DNA-DIRECTED RNA POLYMERASE II SUBUNIT RPB2."" | | | | |  |  |  |  |  |  |
| 13 | HSPA8/HSPA1A | |  |  |  |  |  |  |  |  |  |  |  |
| 10 | POLR2C | 31441 | DNA-DIRECTED RNA POLYMERASE II SUBUNIT RPB3."" | | | | |  |  |  |  |  |  |
| 10 | MED12 | 247334 | MEDIATOR OF RNA POLYMERASE II TRANSCRIPTION SUBUNIT 12."" | | | | | |  |  |  |  |  |
| 9 | SMARCE1 | 46649 | ISOFORM 1 OF SWI/SNF-RELATED MATRIX-ASSOCIATED ACTIN-DEPENDENTREGULATOR OF CHROMATIN SUBFAMILY E MEMBER 1."" | | | | | | | | | |  |
| 9 | HSPA5 | 72422 | HSPA5 PROTEIN."" | |  |  |  |  |  |  |  |  |  |
| 7 | MED24 | 33344 | MEDIATOR COMPLEX SUBUNIT MED24 VARIANT MED24_I3."" | | | | |  |  |  |  |  |  |
| 7 | MED23 | 156194 | ISOFORM 3 OF MEDIATOR OF RNA POLYMERASE II TRANSCRIPTION SUBUNIT 23."" | | | | | | |  |  |  |  |
| 6 | DDX23 | 95583 | PROBABLE ATP-DEPENDENT RNA HELICASE DDX23."" | | | |  |  |  |  |  |  |  |
| 6 | MED21 | 15564 | MEDIATOR OF RNA POLYMERASE II TRANSCRIPTION SUBUNIT 21."" | | | | | |  |  |  |  |  |
| 6 | SULF1 | 9082 | 9 KDA PROTEIN."" | |  |  |  |  |  |  |  |  |  |
| 5 | ARID2 | 197391 | ISOFORM 1 OF AT-RICH INTERACTIVE DOMAIN-CONTAINING PROTEIN 2."" | | | | | |  |  |  |  |  |
| 4 | ACTG1 | 41793 | ACTIN. CYTOPLASMIC 2."" | | |  |  |  |  |  |  |  |  |
| 4 | POLR2H | 17143 | DNA-DIRECTED RNA POLYMERASES I. II. AND III SUBUNIT RPABC3."" | | | | | |  |  |  |  |  |
| 4 | MRPS27 | 49150 | CDNA FLJ54536. HIGHLY SIMILAR TO MITOCHONDRIAL 28S RIBOSOMAL PROTEINS27."" | | | | | | |  |  |  |  |
| 4 | MED11 | 13129 | MEDIATOR OF RNA POLYMERASE II TRANSCRIPTION SUBUNIT 11."" | | | | | |  |  |  |  |  |
| 4 | MED13 | 239318 | MEDIATOR OF RNA POLYMERASE II TRANSCRIPTION SUBUNIT 13."" | | | | | |  |  |  |  |  |
| 3 | MED23 | 157114 | COFACTOR REQUIRED FOR SP1 TRANSCRIPTIONAL ACTIVATION. SUBUNIT 3.130KDA."" | | | | | | |  |  |  |  |
| 2 | TUBB | 49671 | TUBULIN BETA CHAIN."" | |  |  |  |  |  |  |  |  |  |
| 2 | HSPA8 | 70898 | ISOFORM 1 OF HEAT SHOCK COGNATE 71 KDA PROTEIN."" | | | | |  |  |  |  |  |  |
| 2 | POLR2D | 16311 | DNA-DIRECTED RNA POLYMERASE II SUBUNIT RPB4."" | | | | |  |  |  |  |  |  |
| 2 | PBRM1 | 182118 | ISOFORM 5 OF PROTEIN POLYBROMO-1."" | | | |  |  |  |  |  |  |  |
| 2 | NKG_910 | 19245 | ANTIGEN MLAA41 (FRAGMENT)."" | | |  |  |  |  |  |  |  |  |
| 2 | MRPS34 | 25650 | 28S RIBOSOMAL PROTEIN S34. MITOCHONDRIAL."" | | | |  |  |  |  |  |  |  |
| 2 | UCHL5 | 36079 | ISOFORM 2 OF UBIQUITIN CARBOXYL-TERMINAL HYDROLASE ISOZYME L5."" | | | | | |  |  |  |  |  |
| 2 | MED13L | 242602 | MEDIATOR OF RNA POLYMERASE II TRANSCRIPTION SUBUNIT 13-LIKE."" | | | | | |  |  |  |  |  |
| 2 | KIF21A | 6196 | 6 KDA PROTEIN."" | |  |  |  |  |  |  |  |  |  |
| 2 | MED19 | 20431 | ISOFORM 2 OF MEDIATOR OF RNA POLYMERASE II TRANSCRIPTION SUBUNIT 19."" | | | | | | |  |  |  |  |
| 2 | JAK1 | 133277 | TYROSINE-PROTEIN KINASE JAK1."" | | |  |  |  |  |  |  |  |  |
| 2 | MRPS17 | 14502 | 28S RIBOSOMAL PROTEIN S17. MITOCHONDRIAL."" | | | |  |  |  |  |  |  |  |
| 1 | SMARCC2 | 132879 | ISOFORM 1 OF SWI/SNF COMPLEX SUBUNIT SMARCC2."" | | | | |  |  |  |  |  |  |
| 1 | SMARCD2 | 57173 | SWI/SNF-RELATED MATRIX-ASSOCIATED ACTIN-DEPENDENT REGULATOR OFCHROMATIN D2 ISOFORM 2."" | | | | | | | |  |  |  |
| 1 | SMARCB1 | 45050 | CDNA FLJ13963 FIS. CLONE Y79AA1001299. HIGHLY SIMILAR TO HOMO SAPIENSINTEGRASE INTERACTOR 1B PROTEIN."" | | | | | | | | | |  |
| 1 | HSPA9 | 73680 | STRESS-70 PROTEIN. MITOCHONDRIAL."" | | | |  |  |  |  |  |  |  |
| 1 | RASL11B | 27508 | RAS-LIKE PROTEIN FAMILY MEMBER 11B."" | | | |  |  |  |  |  |  |  |
| 1 | CD320 | 28991 | CD320 ANTIGEN."" | |  |  |  |  |  |  |  |  |  |
| 1 | SNRPD2 | 13527 | SMALL NUCLEAR RIBONUCLEOPROTEIN SM D2."" | | | |  |  |  |  |  |  |  |
| 1 | PLBD1 | 63255 | PHOSPHOLIPASE B DOMAIN CONTAINING 1."" | | | |  |  |  |  |  |  |  |
| 1 | PGM3 | 62911 | CDNA FLJ55543. HIGHLY SIMILAR TO PHOSPHOACETYLGLUCOSAMINE MUTASE."" | | | | | | |  |  |  |  |
| 1 | PCDHGA12 | 94477 | ISOFORM 2 OF PROTOCADHERIN GAMMA-C4."" | | | |  |  |  |  |  |  |  |
| 1 | POLR1A | 194811 | DNA-DIRECTED RNA POLYMERASE I SUBUNIT RPA1."" | | | |  |  |  |  |  |  |  |
| 1 | PDE3A | 124979 | CGMP-INHIBITED 3'.5'-CYCLIC PHOSPHODIESTERASE A."" | | | | |  |  |  |  |  |  |
| 1 | MRPS16 | 15345 | 28S RIBOSOMAL PROTEIN S16. MITOCHONDRIAL."" | | | |  |  |  |  |  |  |  |
| 1 | SNRNP40 | 44516 | CDNA FLJ56825. HIGHLY SIMILAR TO WD REPEAT PROTEIN 57."" | | | | |  |  |  |  |  |  |
| 1 | HRNR | 282390 | HORNERIN."" | |  |  |  |  |  |  |  |  |  |
| 1 | GTF3C1 | 241146 | ISOFORM 1 OF GENERAL TRANSCRIPTION FACTOR 3C POLYPEPTIDE 1."" | | | | | |  |  |  |  |  |
| 1 | MGC42105 | 49606 | SERINE/THREONINE-PROTEIN KINASE NIM1."" | | | |  |  |  |  |  |  |  |
| 1 | 4-Sep | 40704 | ISOFORM 1 OF NEURONAL-SPECIFIC SEPTIN-3."" | | | |  |  |  |  |  |  |  |
| 1 | GTF3C3 | 101272 | ISOFORM 1 OF GENERAL TRANSCRIPTION FACTOR 3C POLYPEPTIDE 3."" | | | | | |  |  |  |  |  |
| 1 | ALB | 71704 | PUTATIVE UNCHARACTERIZED PROTEIN ALB."" | | | |  |  |  |  |  |  |  |
| 1 | FASTKD1 | 97411 | ISOFORM 1 OF FAST KINASE DOMAIN-CONTAINING PROTEIN 1."" | | | | |  |  |  |  |  |  |
| 1 | ASPM | 409800 | ISOFORM 1 OF ABNORMAL SPINDLE-LIKE MICROCEPHALY-ASSOCIATED PROTEIN."" | | | | | | |  |  |  |  |
| 1 | LCN1 | 19250 | LIPOCALIN-1."" | |  |  |  |  |  |  |  |  |  |
| 1 | KCNN1 | 59987 | ISOFORM 1 OF SMALL CONDUCTANCE CALCIUM-ACTIVATED POTASSIUM CHANNELPROTEIN 1."" | | | | | | | |  |  |  |
| 1 | C20orf106 | 19603 | UNCHARACTERIZED PROTEIN C20ORF106."" | | | |  |  |  |  |  |  |  |
| 1 | LOC1720 | 11618 | PUTATIVE UNCHARACTERIZED PROTEIN ENSP00000384574."" | | | | |  |  |  |  |  |  |
| 1 | NKG_5627 | 22206 | CONSERVED HYPOTHETICAL PROTEIN."" | | | |  |  |  |  |  |  |  |
| 1 | CTSF | 53366 | CATHEPSIN F."" | |  |  |  |  |  |  |  |  |  |
| 1 | C1orf168 | 82070 | ISOFORM 1 OF UNCHARACTERIZED PROTEIN C1ORF168."" | | | | |  |  |  |  |  |  |
| 1 | MRPS18B | 29396 | 28S RIBOSOMAL PROTEIN S18B. MITOCHONDRIAL."" | | | |  |  |  |  |  |  |  |
| 1 | CDK9 | 42778 | ISOFORM 1 OF CELL DIVISION PROTEIN KINASE 9."" | | | |  |  |  |  |  |  |  |
| 1 | PARP2 | 46914 | CDNA FLJ58629. HIGHLY SIMILAR TO POLY (ADP-RIBOSE) POLYMERASE 2."" | | | | | |  |  |  |  |  |
| 1 | ACAT2 | 44643 | CDNA FLJ53975. HIGHLY SIMILAR TO ACETYL-COA ACETYLTRANSFERASE.CYTOSOLIC."" | | | | | | |  |  |  |  |
