# Supplementary material for: Molecular Architecture of the Human Mediator–RNA Polymerase II–TFIIF Assembly
Source: PLoS Biol. 2011 Mar 29;9(3):e1000603. doi: 10.1371/journal.pbio.1000603 (PMC3066130; doi:10.1371/journal.pbio.1000603)
Supplement: Table S4 — Summary of all proteins identified from mass spectrometry analysis of pol II. Sample isolated as shown in Figure 1B. Note that for structural and functional work completed here, a glycerol gradient sedimentation step was completed in order to isolate the Mediator–pol II or Mediator–pol II–TFIIF assemblies. The pol II sample analyzed using mass spectrometry was evaluated prior to glycerol gradient sedimentation, and thus sub-stoichiometric contaminants are present in this analysis. Nonetheless, it is evident from the spectral counts as well as the pol II silver-stained gel (Figure 1B) that pol II is predominant in this sample even prior to the glycerol gradient purification step. Spectral counts corresponding to keratin were removed from this list. (0.46 MB DOC) [file pbio.1000603.s015.doc]

| **Spectral Counts** | Gene | Prot MW | Protein descriptor | |  | |  |  |  |  |  |  |  |
| --- | --- | --- | --- | --- | --- | --- | --- | --- | --- | --- | --- | --- | --- |
| 1869 | POLR2A | 217206 | DNA-DIRECTED RNA POLYMERASE II SUBUNIT RPB1."" | | | | | |  |  |  |  |  |
| 997 | POLR2B | 133897 | DNA-DIRECTED RNA POLYMERASE II SUBUNIT RPB2."" | | | | | |  |  |  |  |  |
| 479 | POLR2G | 19294 | DNA-DIRECTED RNA POLYMERASE II SUBUNIT RPB7."" | | | | | |  |  |  |  |  |
| 464 | POLR2D | 16311 | DNA-DIRECTED RNA POLYMERASE II SUBUNIT RPB4."" | | | | | |  |  |  |  |  |
| 353 | POLR2C | 31441 | DNA-DIRECTED RNA POLYMERASE II SUBUNIT RPB3."" | | | | | |  |  |  |  |  |
| 188 | POLR2E | 24551 | DNA-DIRECTED RNA POLYMERASES I. II. AND III SUBUNIT RPABC1."" | | | | | | |  |  |  |  |
| 144 | POLR2I | 14523 | DNA-DIRECTED RNA POLYMERASE II SUBUNIT RPB9."" | | | | | |  |  |  |  |  |
| 114 | NKG_8102 | 44602 | CDNA FLJ11352 FIS. CLONE HEMBA1000020. HIGHLY SIMILAR TO TUBULIN BETA-2C CHAIN."" | | | | | | | |  |  |  |
| 105 | ARID1A | 242045 | ISOFORM 1 OF AT-RICH INTERACTIVE DOMAIN-CONTAINING PROTEIN 1A."" | | | | | | |  |  |  |  |
| 101 | POLR2K | 7004 | DNA-DIRECTED RNA POLYMERASES I. II. AND III SUBUNIT RPABC4."" | | | | | | |  |  |  |  |
| 94 | TUBB/NKG_8102 | |  |  | |  |  |  |  |  |  |  |  |
| 68 | POLR2H | 17143 | DNA-DIRECTED RNA POLYMERASES I. II. AND III SUBUNIT RPABC3."" | | | | | | |  |  |  |  |
| 67 | TUBB | 49671 | TUBULIN BETA CHAIN."" | |  | |  |  |  |  |  |  |  |
| 66 | SMARCE1 | 46649 | ISOFORM 1 OF SWI/SNF-RELATED MATRIX-ASSOCIATED ACTIN-DEPENDENTREGULATOR OF CHROMATIN SUBFAMILY E MEMBER 1."" | | | | | | | | | | |
| 65 | SUPT5H | 120499 | ISOFORM 2 OF TRANSCRIPTION ELONGATION FACTOR SPT5."" | | | | | |  |  |  |  |  |
| 65 | DPF2 | 44155 | ZINC FINGER PROTEIN UBI-D4."" | | | |  |  |  |  |  |  |  |
| 56 | GRINL1A | 41740 | ISOFORM 1 OF PROTEIN GRINL1A."" | | | |  |  |  |  |  |  |  |
| 55 | SMARCA4 | 188062 | SWI/SNF-RELATED MATRIX-ASSOCIATED ACTIN-DEPENDENT REGULATOR OFCHROMATIN A4 ISOFORM D."" | | | | | | | | |  |  |
| 51 | TPR | 267293 | NUCLEOPROTEIN TPR."" | |  | |  |  |  |  |  |  |  |
| 47 | SMARCA2 | 179281 | ISOFORM SHORT OF PROBABLE GLOBAL TRANSCRIPTION ACTIVATOR SNF2L2."" | | | | | | | |  |  |  |
| 43 | SMARCC2 | 132879 | ISOFORM 1 OF SWI/SNF COMPLEX SUBUNIT SMARCC2."" | | | | | |  |  |  |  |  |
| 43 | ARID1B | 236123 | ISOFORM 1 OF AT-RICH INTERACTIVE DOMAIN-CONTAINING PROTEIN 1B."" | | | | | | |  |  |  |  |
| 30 | VIM | 53652 | VIMENTIN."" |  | |  |  |  |  |  |  |  |  |
| 26 | HSPA5 | 72422 | HSPA5 PROTEIN."" | |  | |  |  |  |  |  |  |  |
| 25 | MVP | 99327 | MAJOR VAULT PROTEIN."" | |  | |  |  |  |  |  |  |  |
| 24 | RPAP2 | 69509 | ISOFORM 1 OF RNA POLYMERASE II-ASSOCIATED PROTEIN 2."" | | | | | |  |  |  |  |  |
| 23 | NKG_337 | 70962 | 71 KDA PROTEIN."" | |  | |  |  |  |  |  |  |  |
| 23 | ACTL6A | 47461 | ISOFORM 1 OF ACTIN-LIKE PROTEIN 6A."" | | | | |  |  |  |  |  |  |
| 23 | SS18 | 46289 | SSXT PROTEIN."" | |  | |  |  |  |  |  |  |  |
| 22 | SMARCA4/SMARCA2 | |  |  | |  |  |  |  |  |  |  |  |
| 22 | ACTG1/ACTB | |  |  | |  |  |  |  |  |  |  |  |
| 22 | P4HA1 | 60967 | ISOFORM 2 OF PROLYL 4-HYDROXYLASE SUBUNIT ALPHA-1."" | | | | | |  |  |  |  |  |
| 21 | RUVBL2 | 51157 | RUVB-LIKE 2."" | |  | |  |  |  |  |  |  |  |
| 20 | SMARCD2 | 57173 | SWI/SNF-RELATED MATRIX-ASSOCIATED ACTIN-DEPENDENT REGULATOR OFCHROMATIN D2 ISOFORM 2."" | | | | | | | | |  |  |
| 19 | ACTG1 | 41793 | ACTIN. CYTOPLASMIC 2."" | | | |  |  |  |  |  |  |  |
| 19 | KHDRBS1 | 48227 | ISOFORM 1 OF KH DOMAIN-CONTAINING. RNA-BINDING. SIGNAL TRANSDUCTION-ASSOCIATED PROTEIN 1."" | | | | | | | | | |  |
| 18 | RANBP2 | 358199 | E3 SUMO-PROTEIN LIGASE RANBP2."" | | | |  |  |  |  |  |  |  |
| 18 | AKAP8 | 76108 | A-KINASE ANCHOR PROTEIN 8."" | | | |  |  |  |  |  |  |  |
| 18 | VCP | 89322 | TRANSITIONAL ENDOPLASMIC RETICULUM ATPASE."" | | | | |  |  |  |  |  |  |
| 17 | SMARCC1 | 122867 | SWI/SNF COMPLEX SUBUNIT SMARCC1."" | | | | |  |  |  |  |  |  |
| 17 | SF3B1 | 145815 | SPLICING FACTOR 3B SUBUNIT 1."" | | | |  |  |  |  |  |  |  |
| 17 | ANAPC7 | 66855 | ANAPHASE-PROMOTING COMPLEX SUBUNIT 7 ISOFORM A."" | | | | | |  |  |  |  |  |
| 16 | SMARCC2/SMARCC1 | |  |  | |  |  |  |  |  |  |  |  |
| 16 | TUBB/NKG_8102 | |  |  | |  |  |  |  |  |  |  |  |
| 16 | CAD | 242984 | CAD PROTEIN."" | |  | |  |  |  |  |  |  |  |
| 15 | HSPA8 | 70898 | ISOFORM 1 OF HEAT SHOCK COGNATE 71 KDA PROTEIN."" | | | | | |  |  |  |  |  |
| 13 | SMARCB1 | 45050 | CDNA FLJ13963 FIS. CLONE Y79AA1001299. HIGHLY SIMILAR TO HOMO SAPIENSINTEGRASE INTERACTOR 1B PROTEIN."" | | | | | | | | | | |
| 13 | ZCCHC8 | 51130 | ISOFORM 2 OF ZINC FINGER CCHC DOMAIN-CONTAINING PROTEIN 8."" | | | | | | |  |  |  |  |
| 13 | SF3B3 | 135577 | ISOFORM 1 OF SPLICING FACTOR 3B SUBUNIT 3."" | | | | |  |  |  |  |  |  |
| 13 | PRKCSH | 60134 | CDNA FLJ59211. HIGHLY SIMILAR TO GLUCOSIDASE 2 SUBUNIT BETA."" | | | | | | |  |  |  |  |
| 12 | HSPA8/HSPA1A | |  |  | |  |  |  |  |  |  |  |  |
| 12 | SNX9 | 66592 | SORTING NEXIN-9."" | |  | |  |  |  |  |  |  |  |
| 12 | BCL7C | 23365 | ISOFORM 1 OF B-CELL CLL/LYMPHOMA 7 PROTEIN FAMILY MEMBER C."" | | | | | | |  |  |  |  |
| 12 | C1QBP | 31362 | COMPLEMENT COMPONENT 1 Q SUBCOMPONENT-BINDING PROTEIN. MITOCHONDRIAL."" | | | | | | | |  |  |  |
| 11 | TUBB6 | 46702 | CDNA FLJ52712. HIGHLY SIMILAR TO TUBULIN BETA-6 CHAIN."" | | | | | |  |  |  |  |  |
| 11 | ACTB | 13859 | 14 KDA PROTEIN."" | |  | |  |  |  |  |  |  |  |
| 11 | RECQL5 | 111459 | CDNA FLJ61700. HIGHLY SIMILAR TO ATP-DEPENDENT DNA HELICASE Q5."" | | | | | | |  |  |  |  |
| 11 | NCOR1 | 270210 | ISOFORM 1 OF NUCLEAR RECEPTOR COREPRESSOR 1."" | | | | | |  |  |  |  |  |
| 11 | UBAP2L | 103930 | ISOFORM 2 OF UBIQUITIN-ASSOCIATED PROTEIN 2-LIKE."" | | | | | |  |  |  |  |  |
| 11 | TNKS1BP1 | 181782 | ISOFORM 1 OF 182 KDA TANKYRASE-1-BINDING PROTEIN."" | | | | | |  |  |  |  |  |
| 11 | POLR2L | 7645 | DNA-DIRECTED RNA POLYMERASES I. II. AND III SUBUNIT RPABC5."" | | | | | | |  |  |  |  |
| 10 | RANGAP1 | 63542 | RAN GTPASE-ACTIVATING PROTEIN 1."" | | | |  |  |  |  |  |  |  |
| 10 | MYPN | 114695 | ISOFORM 2 OF MYOPALLADIN."" | | | |  |  |  |  |  |  |  |
| 9 | ACTG1/ACTB | |  |  | |  |  |  |  |  |  |  |  |
| 9 | CALU | 37135 | ISOFORM 2 OF CALUMENIN."" | | | |  |  |  |  |  |  |  |
| 8 | CBX3 | 20811 | CHROMOBOX PROTEIN HOMOLOG 3."" | | | |  |  |  |  |  |  |  |
| 7 | CHD7 | 335927 | ISOFORM 1 OF CHROMODOMAIN-HELICASE-DNA-BINDING PROTEIN 7."" | | | | | | |  |  |  |  |
| 7 | POLR2F | 14478 | DNA-DIRECTED RNA POLYMERASES I. II. AND III SUBUNIT RPABC2."" | | | | | | |  |  |  |  |
| 7 | SF3B5 | 10135 | SPLICING FACTOR 3B SUBUNIT 5."" | | | |  |  |  |  |  |  |  |
| 7 | DDX5 | 69148 | PROBABLE ATP-DEPENDENT RNA HELICASE DDX5."" | | | | |  |  |  |  |  |  |
| 6 | HNRNPH1/HNRNPF | |  |  | |  |  |  |  |  |  |  |  |
| 6 | ZC3H18 | 106359 | ISOFORM 1 OF ZINC FINGER CCCH DOMAIN-CONTAINING PROTEIN 18."" | | | | | | |  |  |  |  |
| 6 | HUWE1 | 482148 | 482 KDA PROTEIN."" | |  | |  |  |  |  |  |  |  |
| 6 | UBR5 | 309352 | E3 UBIQUITIN-PROTEIN LIGASE UBR5."" | | | | |  |  |  |  |  |  |
| 6 | SF3B2 | 100228 | SPLICING FACTOR 3B SUBUNIT 2."" | | | |  |  |  |  |  |  |  |
| 6 | ABCC10 | 161629 | ISOFORM 1 OF MULTIDRUG RESISTANCE-ASSOCIATED PROTEIN 7."" | | | | | | |  |  |  |  |
| 6 | THBS1 | 129383 | THROMBOSPONDIN-1."" | |  | |  |  |  |  |  |  |  |
| 6 | INTS1 | 266644 | DKFZP586J0619 PROTEIN."" | | | |  |  |  |  |  |  |  |
| 6 | LMNA | 74139 | ISOFORM A OF LAMIN-A/C."" | | | |  |  |  |  |  |  |  |
| 6 | CCAR1 | 132821 | ISOFORM 1 OF CELL DIVISION CYCLE AND APOPTOSIS REGULATOR PROTEIN 1."" | | | | | | | |  |  |  |
| 6 | LOC646821 | 17136 | SIMILAR TO ACTIN. GAMMA 1."" | | | |  |  |  |  |  |  |  |
| 6 | SEC16A | 251894 | SEC16 HOMOLOG A."" | |  | |  |  |  |  |  |  |  |
| 5 | HNRNPF | 45672 | HETEROGENEOUS NUCLEAR RIBONUCLEOPROTEIN F."" | | | | | |  |  |  |  |  |
| 5 | HNRNPH2 | 49264 | HETEROGENEOUS NUCLEAR RIBONUCLEOPROTEIN H2."" | | | | | |  |  |  |  |  |
| 5 | RFC1 | 128255 | ISOFORM 1 OF REPLICATION FACTOR C SUBUNIT 1."" | | | | | |  |  |  |  |  |
| 5 | GPN1 | 41740 | GPN-LOOP GTPASE 1."" | |  | |  |  |  |  |  |  |  |
| 5 | ZNF451 | 63021 | ISOFORM 3 OF ZINC FINGER PROTEIN 451."" | | | | |  |  |  |  |  |  |
| 5 | CDC16 | 71656 | ISOFORM 1 OF CELL DIVISION CYCLE PROTEIN 16 HOMOLOG."" | | | | | |  |  |  |  |  |
| 5 | G3BP2 | 54121 | ISOFORM A OF RAS GTPASE-ACTIVATING PROTEIN-BINDING PROTEIN 2."" | | | | | | |  |  |  |  |
| 4 | HSPA1A | 70052 | HEAT SHOCK 70 KDA PROTEIN 1."" | | | |  |  |  |  |  |  |  |
| 4 | HSPA9 | 73680 | STRESS-70 PROTEIN. MITOCHONDRIAL."" | | | | |  |  |  |  |  |  |
| 4 | NUMA1 | 236516 | ISOFORM 2 OF NUCLEAR MITOTIC APPARATUS PROTEIN 1."" | | | | | |  |  |  |  |  |
| 4 | DNAJA1 | 44868 | DNAJ HOMOLOG SUBFAMILY A MEMBER 1."" | | | | |  |  |  |  |  |  |
| 4 | GPN3 | 32761 | ISOFORM 1 OF GPN-LOOP GTPASE 3."" | | | |  |  |  |  |  |  |  |
| 4 | SCYL1 | 86312 | ISOFORM 4 OF N-TERMINAL KINASE-LIKE PROTEIN."" | | | | |  |  |  |  |  |  |
| 4 | EEF1D | 31122 | ISOFORM 1 OF ELONGATION FACTOR 1-DELTA."" | | | | |  |  |  |  |  |  |
| 4 | LEPRE1 | 90616 | ISOFORM 3 OF PROLYL 3-HYDROXYLASE 1."" | | | | |  |  |  |  |  |  |
| 4 | C19orf2 | 59832 | ISOFORM 1 OF UNCONVENTIONAL PREFOLDIN RPB5 INTERACTOR."" | | | | | | |  |  |  |  |
| 4 | PRPF39 | 78430 | ISOFORM 1 OF PRE-MRNA-PROCESSING FACTOR 39."" | | | | | |  |  |  |  |  |
| 4 | CHERP | 103573 | ISOFORM 1 OF CALCIUM HOMEOSTASIS ENDOPLASMIC RETICULUM PROTEIN."" | | | | | | |  |  |  |  |
| 4 | WDR5 | 36588 | WD REPEAT-CONTAINING PROTEIN 5."" | | | |  |  |  |  |  |  |  |
| 4 | HDAC1 | 55103 | HISTONE DEACETYLASE 1."" | | | |  |  |  |  |  |  |  |
| 4 | BCL7A | 22810 | ISOFORM 1 OF B-CELL CLL/LYMPHOMA 7 PROTEIN FAMILY MEMBER A."" | | | | | | |  |  |  |  |
| 4 | RBM10 | 110338 | PUTATIVE UNCHARACTERIZED PROTEIN DKFZP686E2459."" | | | | | |  |  |  |  |  |
| 4 | ANKRD52 | 115058 | SERINE/THREONINE-PROTEIN PHOSPHATASE 6 REGULATORY ANKYRIN REPEATSUBUNIT C."" | | | | | | | |  |  |  |
| 4 | C12orf57 | 13178 | PROTEIN C10."" | |  | |  |  |  |  |  |  |  |
| 4 | CDC26 | 9777 | ANAPHASE-PROMOTING COMPLEX SUBUNIT CDC26."" | | | | |  |  |  |  |  |  |
| 4 | ANAPC5 | 85077 | ISOFORM 1 OF ANAPHASE-PROMOTING COMPLEX SUBUNIT 5."" | | | | | |  |  |  |  |  |
| 4 | SAPS1 | 103139 | 103 KDA PROTEIN."" | |  | |  |  |  |  |  |  |  |
| 4 | RCN1 | 38890 | RETICULOCALBIN-1."" | |  | |  |  |  |  |  |  |  |
| 4 | TRAP1 | 80110 | HEAT SHOCK PROTEIN 75 KDA. MITOCHONDRIAL."" | | | | |  |  |  |  |  |  |
| 4 | EIF4G1 | 154933 | EUKARYOTIC TRANSLATION INITIATION FACTOR 4 GAMMA. 1 ISOFORM 4."" | | | | | | |  |  |  |  |
| 3 | MED14 | 160607 | MEDIATOR OF RNA POLYMERASE II TRANSCRIPTION SUBUNIT 14."" | | | | | | |  |  |  |  |
| 3 | HNRNPH1 | 49229 | HETEROGENEOUS NUCLEAR RIBONUCLEOPROTEIN H."" | | | | | |  |  |  |  |  |
| 3 | MED12 | 247334 | MEDIATOR OF RNA POLYMERASE II TRANSCRIPTION SUBUNIT 12."" | | | | | | |  |  |  |  |
| 3 | KIAA1967 | 102902 | ISOFORM 1 OF PROTEIN KIAA1967."" | | | |  |  |  |  |  |  |  |
| 3 | ANKRD28 | 116543 | ISOFORM 1 OF SERINE/THREONINE-PROTEIN PHOSPHATASE 6 REGULATORY ANKYRINREPEAT SUBUNIT A."" | | | | | | | | |  |  |
| 3 | FIP1L1 | 58376 | ISOFORM 3 OF PRE-MRNA 3'-END-PROCESSING FACTOR FIP1."" | | | | | |  |  |  |  |  |
| 3 | TUBA1A | 50136 | TUBULIN ALPHA-1A CHAIN."" | | | |  |  |  |  |  |  |  |
| 3 | MED27 | 35432 | ISOFORM 1 OF MEDIATOR OF RNA POLYMERASE II TRANSCRIPTION SUBUNIT 27."" | | | | | | | |  |  |  |
| 3 | CASC5 | 262632 | ISOFORM 2 OF PROTEIN CASC5."" | | | |  |  |  |  |  |  |  |
| 3 | DYNLL1 | 10366 | DYNEIN LIGHT CHAIN 1. CYTOPLASMIC."" | | | | |  |  |  |  |  |  |
| 3 | SSBP1 | 17260 | SINGLE-STRANDED DNA-BINDING PROTEIN. MITOCHONDRIAL."" | | | | | |  |  |  |  |  |
| 3 | RGPD1 | 12407 | PROTEIN."" |  | |  |  |  |  |  |  |  |  |
| 3 | SEC24B | 140421 | SEC24B PROTEIN."" | |  | |  |  |  |  |  |  |  |
| 3 | PHIP | 206647 | PH-INTERACTING PROTEIN."" | | | |  |  |  |  |  |  |  |
| 3 | KARS | 68048 | LYSYL-TRNA SYNTHETASE."" | | | |  |  |  |  |  |  |  |
| 3 | PCBP2 | 38222 | POLY(RC) BINDING PROTEIN 2 ISOFORM B."" | | | | |  |  |  |  |  |  |
| 3 | C10orf104 | 11667 | UPF0448 PROTEIN C10ORF104."" | | | |  |  |  |  |  |  |  |
| 3 | ADNP | 123563 | ACTIVITY-DEPENDENT NEUROPROTECTOR HOMEOBOX PROTEIN."" | | | | | |  |  |  |  |  |
| 2 | MED1 | 168478 | ISOFORM 1 OF MEDIATOR OF RNA POLYMERASE II TRANSCRIPTION SUBUNIT 1."" | | | | | | | |  |  |  |
| 2 | SMARCC2 | 12331 | 12 KDA PROTEIN."" | |  | |  |  |  |  |  |  |  |
| 2 | ACTBL2 | 42003 | BETA-ACTIN-LIKE PROTEIN 2."" | | | |  |  |  |  |  |  |  |
| 2 | HNRNPH1/HNRNPF | |  |  | |  |  |  |  |  |  |  |  |
| 2 | ALMS1 | 461191 | ALSTROM SYNDROME 1."" | |  | |  |  |  |  |  |  |  |
| 2 | TAF5 | 86830 | ISOFORM LONG OF TRANSCRIPTION INITIATION FACTOR TFIID SUBUNIT 5."" | | | | | | |  |  |  |  |
| 2 | HSPD1 | 61055 | 60 KDA HEAT SHOCK PROTEIN. MITOCHONDRIAL."" | | | | |  |  |  |  |  |  |
| 2 | CLTC | 191615 | ISOFORM 1 OF CLATHRIN HEAVY CHAIN 1."" | | | | |  |  |  |  |  |  |
| 2 | MATR3 | 94623 | MATRIN-3."" |  | |  |  |  |  |  |  |  |  |
| 2 | NUP88 | 83542 | NUCLEAR PORE COMPLEX PROTEIN NUP88."" | | | | |  |  |  |  |  |  |
| 2 | SP1 | 80693 | TRANSCRIPTION FACTOR SP1."" | | | |  |  |  |  |  |  |  |
| 2 | YWHAE | 29174 | 14-3-3 PROTEIN EPSILON."" | | | |  |  |  |  |  |  |  |
| 2 | TP53BP1 | 213574 | ISOFORM 1 OF TUMOR SUPPRESSOR P53-BINDING PROTEIN 1."" | | | | | |  |  |  |  |  |
| 2 | CHCHD2 | 15513 | COILED-COIL-HELIX-COILED-COIL-HELIX DOMAIN-CONTAINING PROTEIN 2.MITOCHONDRIAL."" | | | | | | | | |  |  |
| 2 | RNF219 | 81116 | RING FINGER PROTEIN 219."" | | | |  |  |  |  |  |  |  |
| 2 | WIZ | 178674 | ISOFORM 1 OF PROTEIN WIZ."" | | | |  |  |  |  |  |  |  |
| 2 | RPRD1B | 36900 | REGULATION OF NUCLEAR PRE-MRNA DOMAIN-CONTAINING PROTEIN 1B."" | | | | | | |  |  |  |  |
| 2 | TANC2 | 219650 | ISOFORM 1 OF PROTEIN TANC2."" | | | |  |  |  |  |  |  |  |
| 2 | ALB | 71704 | PUTATIVE UNCHARACTERIZED PROTEIN ALB."" | | | | |  |  |  |  |  |  |
| 2 | SAPS3 | 88952 | ISOFORM 4 OF SERINE/THREONINE-PROTEIN PHOSPHATASE 6 REGULATORY SUBUNIT3."" | | | | | | | |  |  |  |
| 2 | THRAP3 | 108666 | THYROID HORMONE RECEPTOR-ASSOCIATED PROTEIN 3."" | | | | | |  |  |  |  |  |
| 2 | ANKRD40 | 41088 | ANKYRIN REPEAT DOMAIN-CONTAINING PROTEIN 40."" | | | | | |  |  |  |  |  |
| 2 | RAE1 | 40968 | MRNA EXPORT FACTOR."" | |  | |  |  |  |  |  |  |  |
| 2 | HSP90B1 | 92469 | ENDOPLASMIN."" | |  | |  |  |  |  |  |  |  |
| 2 | CCNT1 | 80685 | CYCLIN-T1."" | |  | |  |  |  |  |  |  |  |
| 1 | MED15 | 86753 | ISOFORM 1 OF MEDIATOR OF RNA POLYMERASE II TRANSCRIPTION SUBUNIT 15."" | | | | | | | |  |  |  |
| 1 | MED31 | 15805 | MEDIATOR OF RNA POLYMERASE II TRANSCRIPTION SUBUNIT 31."" | | | | | | |  |  |  |  |
| 1 | PRPF6 | 106925 | PRE-MRNA-PROCESSING FACTOR 6."" | | | |  |  |  |  |  |  |  |
| 1 | MED30 | 20277 | MEDIATOR OF RNA POLYMERASE II TRANSCRIPTION SUBUNIT 30."" | | | | | | |  |  |  |  |
| 1 | PJA2 | 78242 | ISOFORM 1 OF E3 UBIQUITIN-PROTEIN LIGASE PRAJA2."" | | | | | |  |  |  |  |  |
| 1 | PIP5K1A | 58119 | PHOSPHATIDYLINOSITOL-4-PHOSPHATE 5-KINASE. TYPE I. ALPHA ISOFORM 3."" | | | | | | | |  |  |  |
| 1 | EIF3J | 29062 | EUKARYOTIC TRANSLATION INITIATION FACTOR 3 SUBUNIT J."" | | | | | |  |  |  |  |  |
| 1 | KIAA1429 | 202025 | ISOFORM 1 OF PROTEIN VIRILIZER HOMOLOG."" | | | | |  |  |  |  |  |  |
| 1 | SUPT4H1 | 13193 | TRANSCRIPTION ELONGATION FACTOR SPT4."" | | | | |  |  |  |  |  |  |
| 1 | DDB1 | 126968 | DNA DAMAGE-BINDING PROTEIN 1."" | | | |  |  |  |  |  |  |  |
| 1 | HBA1 | 15258 | HEMOGLOBIN SUBUNIT ALPHA."" | | | |  |  |  |  |  |  |  |
| 1 | CDKL5 | 115538 | CYCLIN-DEPENDENT KINASE-LIKE 5."" | | | |  |  |  |  |  |  |  |
| 1 | HNRNPC | 32338 | ISOFORM C1 OF HETEROGENEOUS NUCLEAR RIBONUCLEOPROTEINS C1/C2."" | | | | | | |  |  |  |  |
| 1 | CDKN2AIP | 61125 | CDKN2A INTERACTING PROTEIN."" | | | |  |  |  |  |  |  |  |
| 1 | PPP1R13L | 44106 | PROTEIN PHOSPHATASE 1. REGULATORY (INHIBITOR) SUBUNIT 13 LIKE. ISOFORMCRA_B."" | | | | | | | |  |  |  |
| 1 | USP9X | 290463 | UBIQUITIN SPECIFIC PROTEASE 9. X-LINKED ISOFORM 4."" | | | | | |  |  |  |  |  |
| 1 | TRIM8 | 61489 | TRIPARTITE MOTIF-CONTAINING PROTEIN 8."" | | | | |  |  |  |  |  |  |
| 1 | HNRNPM | 77516 | ISOFORM 1 OF HETEROGENEOUS NUCLEAR RIBONUCLEOPROTEIN M."" | | | | | | |  |  |  |  |
| 1 | SKIV2L2 | 117805 | SUPERKILLER VIRALICIDIC ACTIVITY 2-LIKE 2."" | | | | |  |  |  |  |  |  |
| 1 | RING1 | 39146 | ISOFORM 2 OF E3 UBIQUITIN-PROTEIN LIGASE RING1."" | | | | | |  |  |  |  |  |
| 1 | NFAT5 | 165763 | ISOFORM C OF NUCLEAR FACTOR OF ACTIVATED T-CELLS 5."" | | | | | |  |  |  |  |  |
| 1 | KPNA2 | 57862 | IMPORTIN SUBUNIT ALPHA-2."" | | | |  |  |  |  |  |  |  |
| 1 | ZNF295 | 118870 | ZINC FINGER PROTEIN 295."" | | | |  |  |  |  |  |  |  |
| 1 | RUVBL1 | 50228 | ISOFORM 1 OF RUVB-LIKE 1."" | | | |  |  |  |  |  |  |  |
| 1 | CPS1 | 164939 | ISOFORM 1 OF CARBAMOYL-PHOSPHATE SYNTHASE [AMMONIA]. MITOCHONDRIAL."" | | | | | | | |  |  |  |
| 1 | JAKMIP3 | 48807 | ISOFORM 2 OF JANUS KINASE AND MICROTUBULE-INTERACTING PROTEIN 3."" | | | | | | |  |  |  |  |
| 1 | TJP1 | 195459 | ISOFORM LONG OF TIGHT JUNCTION PROTEIN ZO-1."" | | | | | |  |  |  |  |  |
| 1 | P4HA2 | 60902 | ISOFORM IIB OF PROLYL 4-HYDROXYLASE SUBUNIT ALPHA-2."" | | | | | |  |  |  |  |  |
| 1 | EHMT2 | 132370 | ISOFORM 1 OF HISTONE-LYSINE N-METHYLTRANSFERASE. H3 LYSINE-9 SPECIFIC3."" | | | | | | | |  |  |  |
| 1 | CDC27 | 91867 | CELL DIVISION CYCLE PROTEIN 27 HOMOLOG."" | | | | |  |  |  |  |  |  |
| 1 | EIF3CL | 105344 | EUKARYOTIC TRANSLATION INITIATION FACTOR 3 SUBUNIT C."" | | | | | |  |  |  |  |  |
| 1 | DNAJA2 | 45746 | DNAJ HOMOLOG SUBFAMILY A MEMBER 2."" | | | | |  |  |  |  |  |  |
| 1 | POLR2J | 14131 | RPB11A PROTEIN."" | |  | |  |  |  |  |  |  |  |
| 1 | TPX2 | 85653 | TARGETING PROTEIN FOR XKLP2."" | | | |  |  |  |  |  |  |  |
| 1 | HNRNPUL2 | 85105 | HETEROGENEOUS NUCLEAR RIBONUCLEOPROTEIN U-LIKE PROTEIN 2."" | | | | | | |  |  |  |  |
| 1 | TOR1AIP1 | 47578 | TORSIN A INTERACTING PROTEIN 1."" | | | |  |  |  |  |  |  |  |
| 1 | RCN2 | 36876 | RETICULOCALBIN-2."" | |  | |  |  |  |  |  |  |  |
| 1 | SEC23IP | 111076 | ISOFORM 1 OF SEC23-INTERACTING PROTEIN."" | | | | |  |  |  |  |  |  |
| 1 | CPSF3 | 77486 | CLEAVAGE AND POLYADENYLATION SPECIFICITY FACTOR SUBUNIT 3."" | | | | | | |  |  |  |  |
| 1 | PARP4 | 192589 | POLY [ADP-RIBOSE] POLYMERASE 4."" | | | |  |  |  |  |  |  |  |
| 1 | NKG_9227 | 46984 | 47 KDA PROTEIN."" | |  | |  |  |  |  |  |  |  |
| 1 | HSPB1 | 22783 | HEAT SHOCK PROTEIN BETA-1."" | | | |  |  |  |  |  |  |  |
| 1 | WTAP | 44244 | ISOFORM 1 OF PRE-MRNA-SPLICING REGULATOR WTAP."" | | | | | |  |  |  |  |  |
| 1 | RAVER1 | 79579 | RAVER1."" |  | |  |  |  |  |  |  |  |  |
| 1 | NKG_9737 | 34693 | PROTEIN."" |  | |  |  |  |  |  |  |  |  |
| 1 | RFWD3 | 85094 | RING FINGER AND WD REPEAT DOMAIN-CONTAINING PROTEIN 3."" | | | | | |  |  |  |  |  |
| 1 | BAT2D1 | 317208 | ISOFORM 7 OF BAT2 DOMAIN-CONTAINING PROTEIN 1."" | | | | | |  |  |  |  |  |
| 1 | NOTCH1 | 272462 | PUTATIVE UNCHARACTERIZED PROTEIN NOTCH1."" | | | | |  |  |  |  |  |  |
| 1 | MKI67 | 358694 | ISOFORM LONG OF ANTIGEN KI-67."" | | | |  |  |  |  |  |  |  |
| 1 | MTA2 | 75023 | METASTASIS-ASSOCIATED PROTEIN MTA2."" | | | | |  |  |  |  |  |  |
| 1 | RBBP4 | 47656 | HISTONE-BINDING PROTEIN RBBP4."" | | | |  |  |  |  |  |  |  |
| 1 | SETD2 | 149254 | 149 KDA PROTEIN."" | |  | |  |  |  |  |  |  |  |
| 1 | NAV2 | 161920 | ISOFORM 5 OF NEURON NAVIGATOR 2."" | | | | |  |  |  |  |  |  |
| 1 | MAP7 | 75314 | ISOFORM 3 OF ENSCONSIN."" | | | |  |  |  |  |  |  |  |
| 1 | HSP90AA1 | 98161 | ISOFORM 2 OF HEAT SHOCK PROTEIN HSP 90-ALPHA."" | | | | | |  |  |  |  |  |
| 1 | TBCA | 12855 | TUBULIN-SPECIFIC CHAPERONE A."" | | | |  |  |  |  |  |  |  |
| 1 | SRCAP | 337415 | ISOFORM 2 OF HELICASE SRCAP."" | | | |  |  |  |  |  |  |  |
